# Experimental and analytical study on axial behaviour of square corrugated concrete filled single and double skin tube stub columns

**DOI:** 10.1038/s41598-025-85585-z

**Published:** 2025-01-16

**Authors:** Aya Mohsen Handousa, Mohamed Abdellatief, Fikry Abdo Salem, Nabil Mahmoud, Mohamed Ghannam

**Affiliations:** 1 Department of Structural Engineering, Higher Future Institute for Engineering and Technology in Mansoura, Mansoura, Egypt; 2 Department of Structural Engineering, Faculty of Engineering, Mansoura University, Mansoura, Egypt

**Keywords:** Corrugated concrete-filled steel tubes, Corrugated-columns, Square CFDST columns, Ultimate axial strength, Internal square tubes, Civil engineering, Power stations

## Abstract

Concrete-filled double-skin steel tubular (CFDST) columns have become widely utilized in building construction and bridges, thanks to their exceptional structural capabilities. Therefore, this study investigates the axial compressive behavior of square CFDST columns. The study aims to explore the influence of external and internal plate shapes (flat or corrugated plates) and different widths of internal steel tubes on the axial compressive behavior. The effects of varying internal widths of the internal steel tube (60 mm, 116 mm, and 160 mm) on the performance of CFDST columns were examined. Additionally, the study compared the performance of concrete-filled steel tubular (CFST) and CFDST columns with external flat or corrugated plates. The findings indicated that incorporating internal corrugated plates notably improved both the load-carrying capacity and ductility of the specimens. Notably, CFDST columns featuring corrugated internal plates (116 mm width) exhibited strength enhancements of 25.3% and 7.4% compared to internal widths of 160 mm and 60 mm, respectively. Furthermore, the study proposed two machine-learning models, namely Artificial Neural Network (ANN) and Gaussian Process Regression (GPR), to estimate the ultimate compressive strength of square CFDST columns. The findings indicated that the GPR model outperformed the ANN model in predicting the bearing capacity of square CFDST columns. Additionally, the Shapley Additive Explanation technique was employed for feature analysis. The outcomes of this analysis revealed that parameters such as section width and concrete strength positively influence the compressive strength index.

**Supplementary Information:**

The online version contains supplementary material available at 10.1038/s41598-025-85585-z.

## Introduction

Concrete-filled double-skin steel tube (CFDST) columns actually offer several advantages in construction, especially in seismic-prone areas^1^. Their combination of low production costs, high stiffness, and strength makes them increasingly attractive for various structural applications^1, 2^. In seismic regions, the superior performance of CFDST columns during earthquakes is particularly noteworthy. The confinement provided by the external and internal steel tubes effectively enhances the ductility and strength of the concrete infill^2^. This characteristic makes CFDST columns suitable for use in seismically resilient buildings such as bridges and tall piers^3, 4^. As urbanization continues and the demand for high-rise buildings grows, there’s a need for structures that can accommodate long spans and heavy loads. However, traditional frame structures and foundations may become too heavy to withstand seismic forces effectively, which confirms the CFDST columns present a promising solution^1, 2^. CFDST columns^5, 6^, typically exhibit an axial load-carrying capacity that is 10–30% higher than the combined strengths of their components. Recently, instances of yielding, fracture, and significant bending in CFDST columns and steel structures have been investigated^6, 7^. Various factors, including fire, corrosion, pollution, and aging, contribute to these deteriorations. Additionally, even structures that withstand earthquakes without collapsing sometimes suffer considerable damage. Consequently, there’s a pressing need to reinforce these structures to withstand their designated loads or potentially upgrade them to endure even higher stresses^7–9^. For instance, Uenaka^10^ explored the effect of geometric shape parameters on the failure modes and ultimate capacity of CFDST columns. They found a strong correlation between failure modes and the ratio of internal width to external diameter of shape, which also determined the ultimate capacity of the columns. Wang et al.^11^ established a nonlinear finite element analysis model to study the compressive behavior and design methods of a novel CFDST short column with external stainless-steel tubes. They observed a degradation in the ultimate strength of the composite column with an increase in the hollow ratio of samples.

Further experiments were carried out on concentrically loaded square CFDST columns^12, 13^. Experimental research systematically investigated the impact of concrete strength, eccentricity ratio, and section hollow ratio on the strength, deformation, and ductility of specimens. The findings showed that the ultimate strength of the column was primarily affected by the concrete strength, with the section hollow ratio also playing a significant role^12^. The ductility of the column exhibited a positive correlation with the section hollow ratio, which contrasted entirely with the effect of concrete strength. However, the eccentricity ratio had little impact on the ductility of the column. Zhang et al.^14^, Yan et al.^15^, Uenaka^10^ explored the influence of steel internal tubes on the mechanical behavior of CFDST columns under axial compression. Their studies indicated that properly configured CFDST columns could outperform concrete-filled steel tube (CFST) columns. Even with high-strength concrete, the internal tube exhibited behavior consistent with the shell concrete, effectively minimizing failure mechanisms in CFDST structures.

Corrugated plates offer several advantages such as being lightweight, economical, and possessing significantly higher load-carrying capacities compared to flat plates, which has spurred research interest since their introduction^7, 16, 17^. Smith et al.^17^ conducted tests on 5 corrugated steel pipes filled with grout under two-point loading, revealing substantial improvements in strength and stiffness compared to hollow corrugated steel pipes. Han et al.^16^ explored the axial and lateral behavior of CFDST columns with corrugated internal tubes. Their experimental findings demonstrated that CFDST with corrugated internal tubes exhibited superior energy dissipation capacity. Additionally, the corrugated shape provides continuous stiffening, enabling the use of internal plates. Corrugated plates can be easily bent in one direction while maintaining rigidity in the other direction. Moreover, fabrication costs for elements using corrugated panels are typically lower than those using stiffened plates. Compression tests on square hollow columns constructed from corrugated plates by Nassirnia et al.^18^ and Farahi et al.^19, 20^ further supported these findings. Their experiments indicated that corrugated plates enhance local and global stability, ductility, and energy absorption capacity in structural applications. Besides that, Han et al.^21^conducted a study analyzing the structural characteristics of composite columns, examining 80 samples, of which six were configured as CFDST columns with specific dimensions and material properties. Each column featured an external circumference of 220 mm, internal circumference of 159 mm, and a steel tube thickness of 3.75 mm. The study introduced a novel equation to estimate the axial compression capacity of CFDST columns, which demonstrated good agreement with empirical observations. Recently, Aghamaleki et al.^22^ employed machine learning (ML) methods to predict the axial capacity of CFDST columns under hydrostatic pressure. This approach reflects advancements in composite member technology and the exploration of innovative methodologies to meet modern construction requirements. Current design codes, such as ACI, Eurocode-4, and AISC, GB 50,936 − 2014, AIJ (1997) and CECS 159:2004 offer mathematical formulations for predicting the axial compression strength of CFDST columns, aiming for simplicity and accessibility through the use of streamlined variables. However, discrepancies often emerge when these equations are applied to real-world scenarios, raising concerns about their reliability. These concerns are particularly relevant given the numerous factors that influence the axial compression strength of CFDST columns.

Based on the discussion above, this study aims to explore the behavior of axially loaded square CFDST columns with corrugated and flat plates, considering different ratios of external and internal width of the columns, the effect of the shape of corrugations on the internal and external skins, and different hollow ratio (χ). The load-deformation behavior of square CFDST columns will be analyzed, focusing on axial load versus longitudinal strain in both the external steel tube and internal steel tube. Additionally, experimental results will be compared with design standards from Eurocode-4, ACI-318, AISC-360, GB 50,936 − 2014, AIJ (1997) and CECS 159:2004 and the equation proposed by Ding et al.^23^ and proposed equation by Hasan et al.^24^.To explore the relationship between input features and strength capacity as output variable, two robust ML methodologies, Artificial Neural Network (ANN) and Gaussian Process Regression (GPR) models, are employed to predict the strength capacity of square CFDST columns.

## Experimental program

### Test samples

**Fig. 1:**
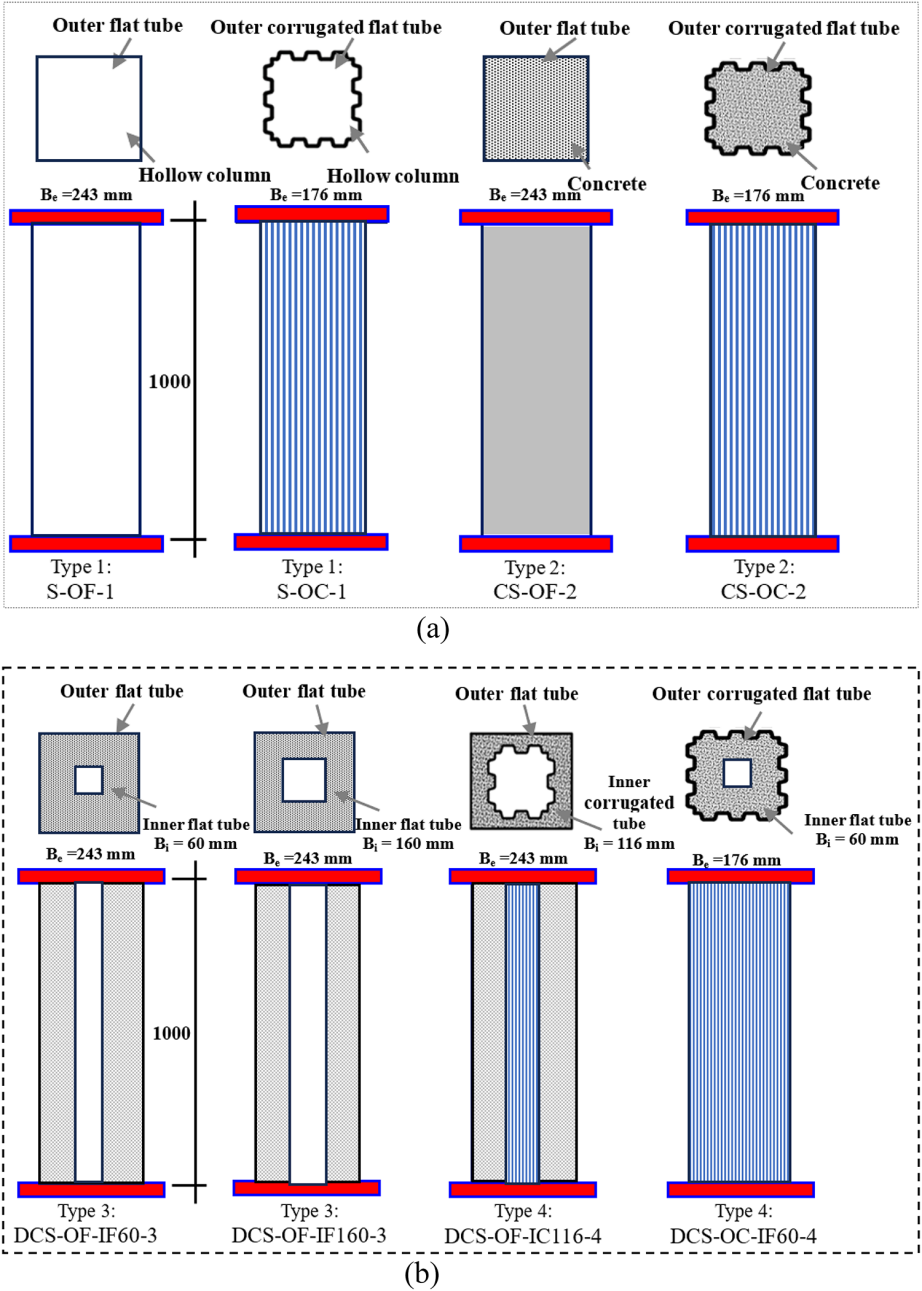
Schematics of square CFDST columns (unit: mm).

The current study assessed the structural behavior of square CFDST columns subjected to axial compression, incorporating corrugated and flat plates, through a series of experiments. Eight square columns were constructed using corrugated plates and flat plates, as presented in Fig. 1. Among them, two columns consisted of hollow steel tubes with flat and corrugated plates (Fig. 1a), respectively. Two columns were filled with concrete as CFST columns (Fig. 1b), while the remaining four columns comprised external and internal steel skins with sandwiched concrete between them as CFDST columns. The external and internal skins varied by incorporating corrugated and flat plates while maintaining a constant area of steel. In this phase, the experimental work was designed to explore the impact of corrugation shape on the performance of hollow steel tube, CFST columns, and CFDST columns. Additionally, the influence of corrugation shape on the internal tube was examined to illustrate the differences in response when using corrugated plates instead of flat plates, as well as the disparities between utilizing corrugation shapes on the external and internal tubes. It’s noteworthy that, to analyze the effect of corrugated plate geometry on the performance of the proposed CFDST columns, the steel area of all columns was kept constant.

**Fig. 2:**
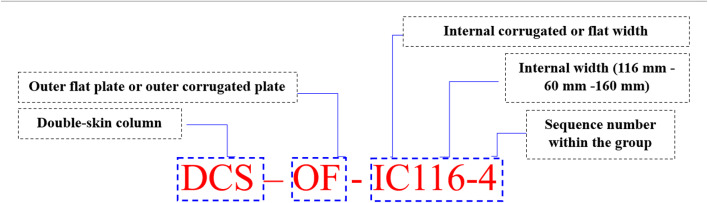
Nomenclature of samples.

As previously indicated and detailed in Table 1, a thorough investigation included 8 columns tested under static axial loading. These columns were classified into four groups, illustrated in Fig. 1. Each specimen shared a common external steel tube area (A_se_) of 2880 mm² and a height (L) of 1000 mm. According to Group 1 (without concrete), the S-OF-1 column has an external width (B_e_) of 243 mm with a thickness (t_o_) of 3 mm, while the S-OC-1 has Be of 176 mm. Group 2 (without concrete) contains CS-OF-2 and CS-OC-2. On the other hand, Group 3 contains DCS-OF-IF60-3 and DCS-OF-IF160-3, which represent CS-OF-2 with different internal flat plate widths (B_i_) of 60 and 160 mm, respectively. Finally, in Group 4, the DCS-OF-IC116-4 column features an external flat plate with a B_e_ of 243 mm and an internal corrugated B_i_ of 116 mm. Conversely, the DCS-OC-IF60-4 column utilizes an external corrugated plate with a B_e_ of 176 mm and an internal corrugated B_i_ of 60 mm. Ratios of external width to external thickness (B_e_/t_o_) were set at 58.67 and 81, while internal width to internal thickness (B_i_/t_i_) ratios of 20, 38.68, and 53.33 were employed. The column’s length-to-external width ratio (L/B_e_) remained consistent, ranging between 4.1 and 5.68, while the length-to-external width ratio (L/B_i_) varied from 6.25 to 16.67. For clarity, Fig. 2 provides labels for the specimens. To illustrate, let’s take “DCS-OF-IC116-4” from Table 1 as an example. The designation “DCS” indicates a double-skin column comprising an external flat steel tube and an internal corrugated steel tube. Also, S-OF indicates “steel columns with external flat plates,” and CS-OC indicates “concrete-filled columns with external corrugated plates.” The number “4” represents the sequence number of the specimen within its group. It’s worth noting that the number “116” signifies the width of the internal hollow. Additionally, the hollow ratio (χ) was ranged from 0 to 0.66, which can be determined as follows in Eq. (1):

1 $$\:{\upchi\:}\:=\:\frac{{B}_{i}}{{B}_{o}-2{t}_{o}}$$

**Table 1:** Details of test specimens.

Column	External tube dimension (mm)		Internal tube dimension (mm)			χ	$\:{B}_{e}/{t}_{e}$	$\:{B}_{i}/{t}_{i}$	*L* mm	L/B_e_	L/B_i_	ξ	A_c_, mm^2^
	B_e_	$\:{t}_{e}$	$\:{B}_{i}$	$\:{t}_{i}$	A_si_								
S-OF-1	243	3	–	–	–	–	81	–	1000	4.11	–	–	–
S-OC-1	176						58.67			5.68			
CS-OF-2	243						81			4.11		0.44	56,169
CS-OC-2	176						58.67			5.68		0.74	35,518
DCS-OF-IF60-3	243		60	3	684	0.25	81	20		4.11	16.67	0.47	52,569
DCS-OF-IF160-3	243		160		1884	0.66	58.67	53.33		4.11	6.25	0.8	30,569
DCS-OF-IC116-4	243		116		1884	0.48	81	20		5.68	16.67	0.75	42,703
DCS-OC-IF60-4	176		60		684	0.34	58.67	38.68		4.11	8.62	0.58	34,834

### Properties of materials

#### Steel material

**Fig. 3:**
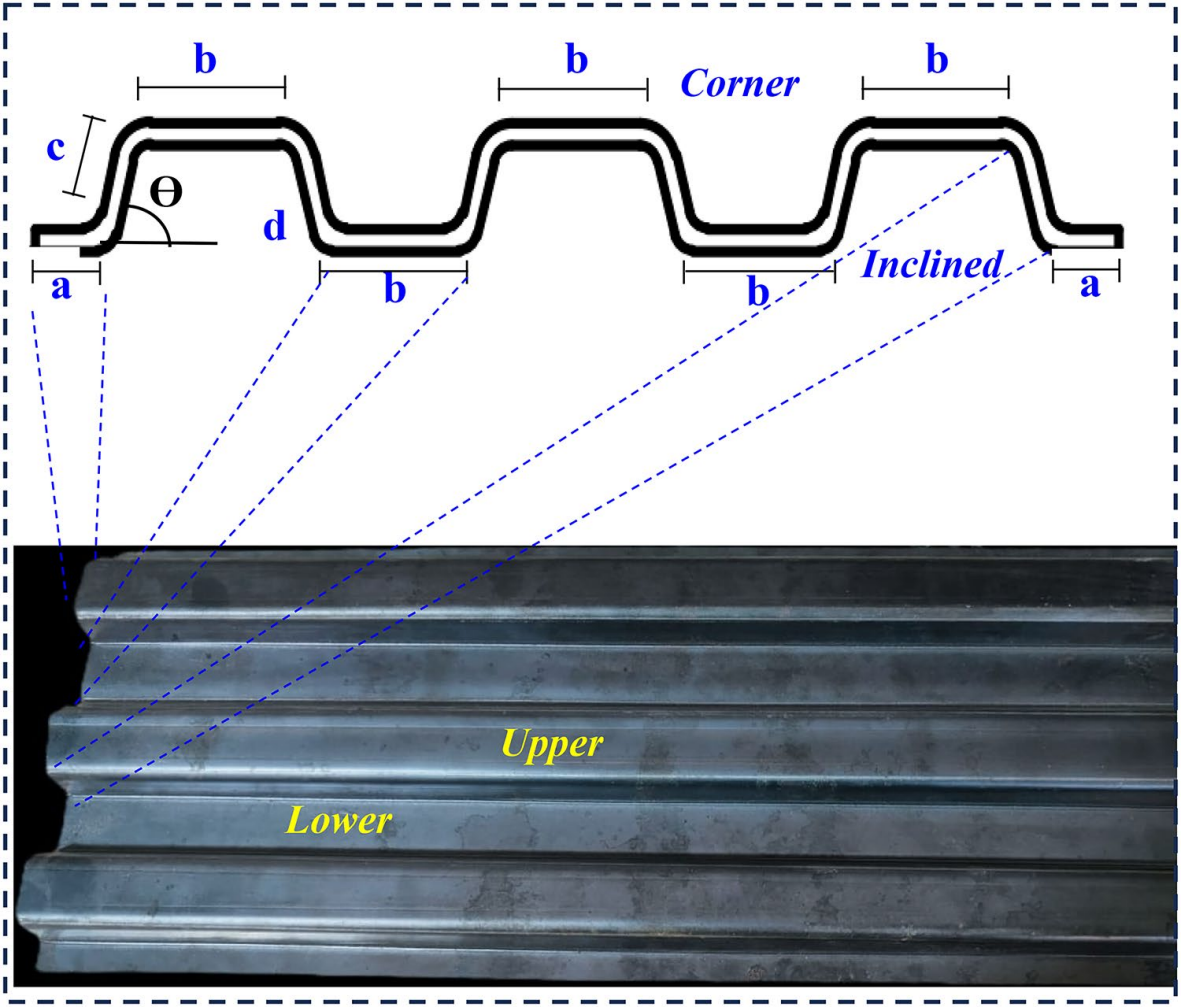
The geometry of the cross-section of corrugated plate.

**Fig. 4:**
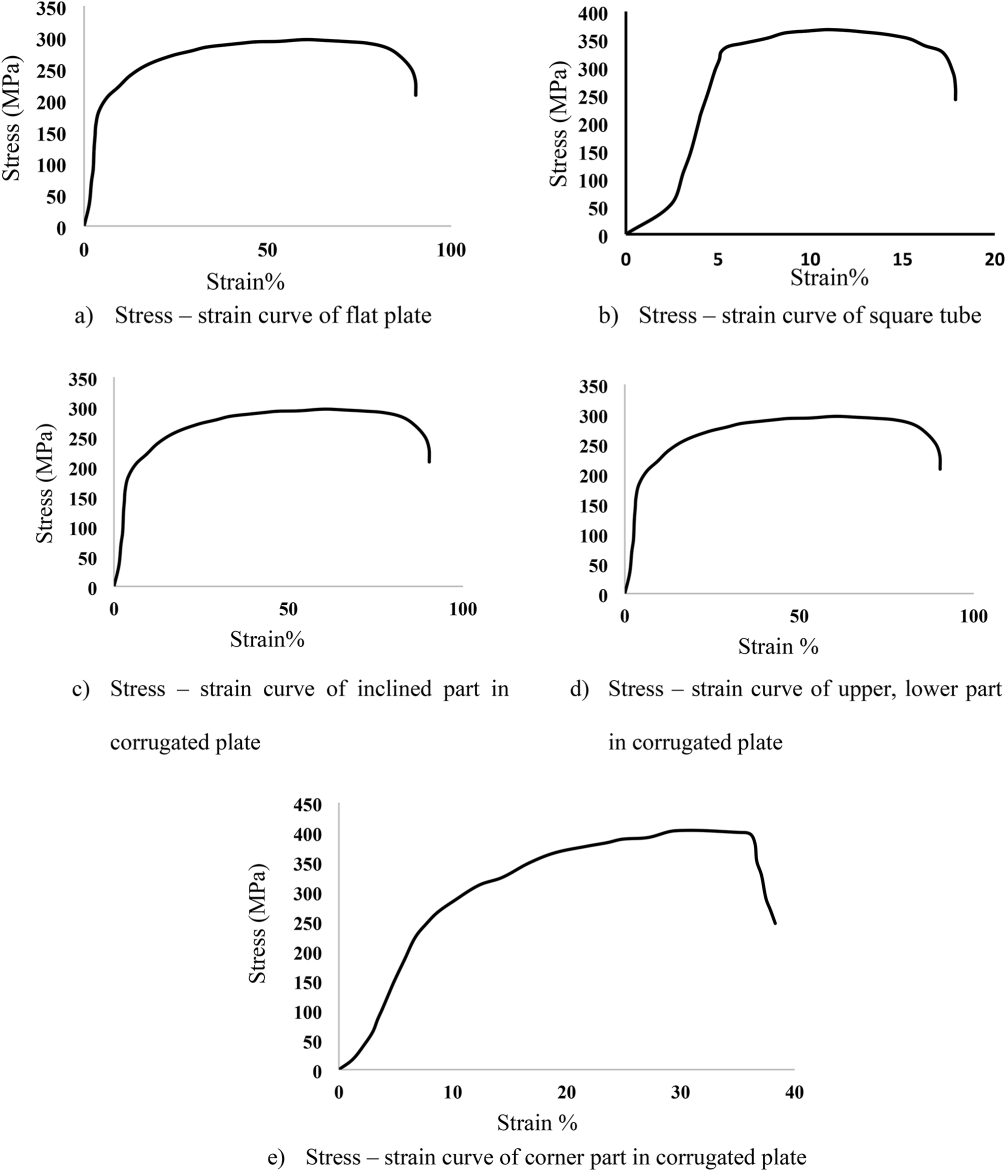
Stress – strain curve of steel section.

The column specimen consisted of two steel tubes with sandwiched concrete between them. The external tube of flat columns was formed from four straight sides tied together by welds and the external tube of corrugated columns was formed from four corrugated plates welded at corners. The internal square tube was either a hot-rolled or built-up section formed by welding four flat or corrugated. Three different types of steel materials were adopted in the tests for the flat plate square tube and corrugation plates, as presented in Table 2. The compressive behavior of the CFDST columns affected by the geometry of the corrugated plate, in which the effects of the inclination angle (ϴ), the inclination distance (c) and corrugation height (h) were considered, as presented in Fig. 3. The corrugated plates in this study were formed from Grade 240 mild-steel flat plates measuring 243 mm in width, 1000 mm in length, and 3 mm in thickness. These flat plates were cold-formed into corrugated plates using the press-braking method, as depicted in Fig. 3. This cold-forming process influences the mechanical characteristics of the folded plates, particularly at the corners of the corrugations. It is anticipated that this process will lead to a reduction in ductility while increasing both yield strength and ultimate strength in these specific areas^25^. Additionally, to determine the steel material properties for each type of steel, tensile coupons were cut with dimensions in accordance with the Australian Standard AS 1391. The average yield stresses for the corrugated steel samples were 202 MPa, 202 MPa, 203 MPa, and 263 MPa, as depicted in Table 3; Fig. 4.

**Table 2:** Properties of the utilised steel materials.

Steel type	Yield strength [MPa]	Ultimate strength [MPa]	Elastic modulus [MPa]
Flat plate	203	269	2 × 10^5^
Square tube	336	368	1.97 × 10^5^

**Table 3:** Geometric properties of the corrugated plates and its corrugated steel materials properties.

Geometric properties (dimensions)				
a (mm)	b (mm)	c (mm)	h (mm)	ϴ
12.5	25	15.33	15	75^0^

Properties of the corrugated steel materials				
Properties [MPa]	Yield strength	Ultimate strength	Elastic modulus	
Upper	202	269	2 × 10^5^	
Lower	202	269	1.97 × 10^5^	
Inclined	203	269	2 × 10^5^	
Corner	263	401	2 × 10^5^	

#### Concrete material

**Fig. 5:**
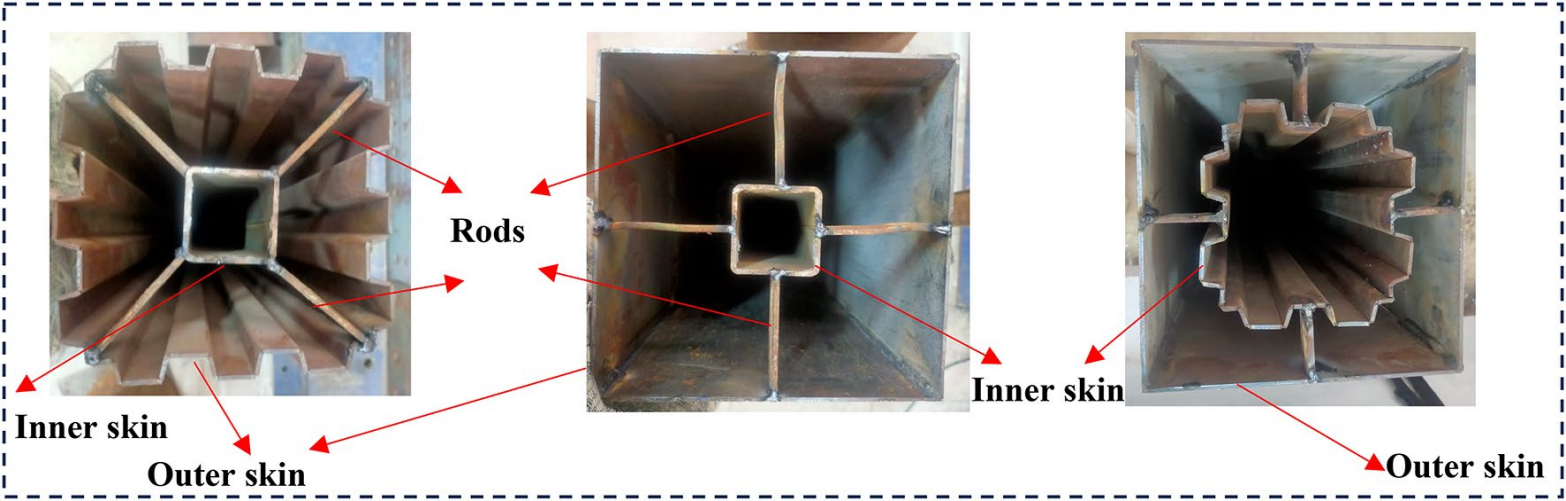
The specimens prepared to cast concrete.

For evaluating the compressive strength (*f*_c_) of concrete grade M25, three cubic concrete samples measuring 150 × 150 × 150 mm^3^ were prepared, as presented in Table 4. The same grade of concrete was utilized to fill the sandwiched area of the CFDST columns. The samples were fabricated in 5 layers of casting, with each layer compacted using a vibrator. Subsequently, the specimens were cured at room temperature for a duration of 28 days. Prior to insertion into the universal testing machine (UTM), the specimen ends underwent preparation. At the top of the columns, four mild steel rods were utilized to secure the internal tube to the external tube, as illustrated in Fig. 5. Subsequently, an upper cover steel plate was employed to cap the column’s top through welding, specifically onto the external steel tube. This measure ensured that both the steel tubes and the sandwiched concrete functioned collaboratively in distributing stress during the loading process (Fig. 5).

**Table 4:** Properties of the prepared concrete.

Normal concrete (MPa)	Water/cement ratio	Mix proportion (by weight%)			
		Cement	Water	Fine aggregate	Coarse aggregate
25	0.55	1.0	0.55	1.892	3.14

### Proposed novel composite columns

This paper introduces three novel types of composite columns: CS-OC-2, DCS-OF-IC116-4, and DCS-OC-IF60-4. Their primary distinction lies in the external corrugated steel tube design in both CS-OC-2 and DCS-OC-IF60-4, while DCS-OF-IC116-4 employs internal corrugated steel tubes. As explored by previous studies^26, 27^, the internal steel tube primarily serves as an internal boundary for the enclosed concrete, with the strength properties of a CFDST chiefly influenced by the confinement factor (ξ). In this investigation, CFDST were examined with six distinct confinement factors (in the range of ξ = 0.44 to 0.80), as presented in Table 1. The mathematical model for the ξ aligns with previous studies^26, 28^, as indicated in Eq. (2):

2 $$\:{\upxi\:}\hspace{0.17em}=\hspace{0.17em}{\upalpha\:}\:\frac{{f}_{ye}}{{f}_{cu}}$$

Here, *f*_ye_ represents the steel yield strength, *f*_cu_ denotes the strength of concrete, and α represents the nominal steel ratio, calculated by α = A_se_/A_c_. A_se_ represents the cross-sectional area of the external steel plate. A_c_ signifies the nominal cross-sectional area of concrete, determined through Eq. (3).

3 $$\:\text{A}\text{c}\:\left(\text{f}\text{o}\text{r}\:\text{t}\text{h}\text{e}\:\text{c}\text{o}\text{r}\text{r}\text{u}\text{g}\text{a}\text{t}\text{e}\text{d}\:\text{p}\text{l}\text{a}\text{t}\text{e}\right)\hspace{0.17em}=\hspace{0.17em}\text{B}\text{e}2\:+\:\text{n}\:\left(\:\frac{b+d}{2}*h\right)$$

Where, A_c_ represents the cross-sectional area of the external corrugated steel plate, B_e_ is the external width of corrugated steel plate, n is the number of corrugated faces, b, d, and h is the width, depth, and corrugation height of the corrugated faces.

### Experimental setup and measurements

**Fig. 6:**
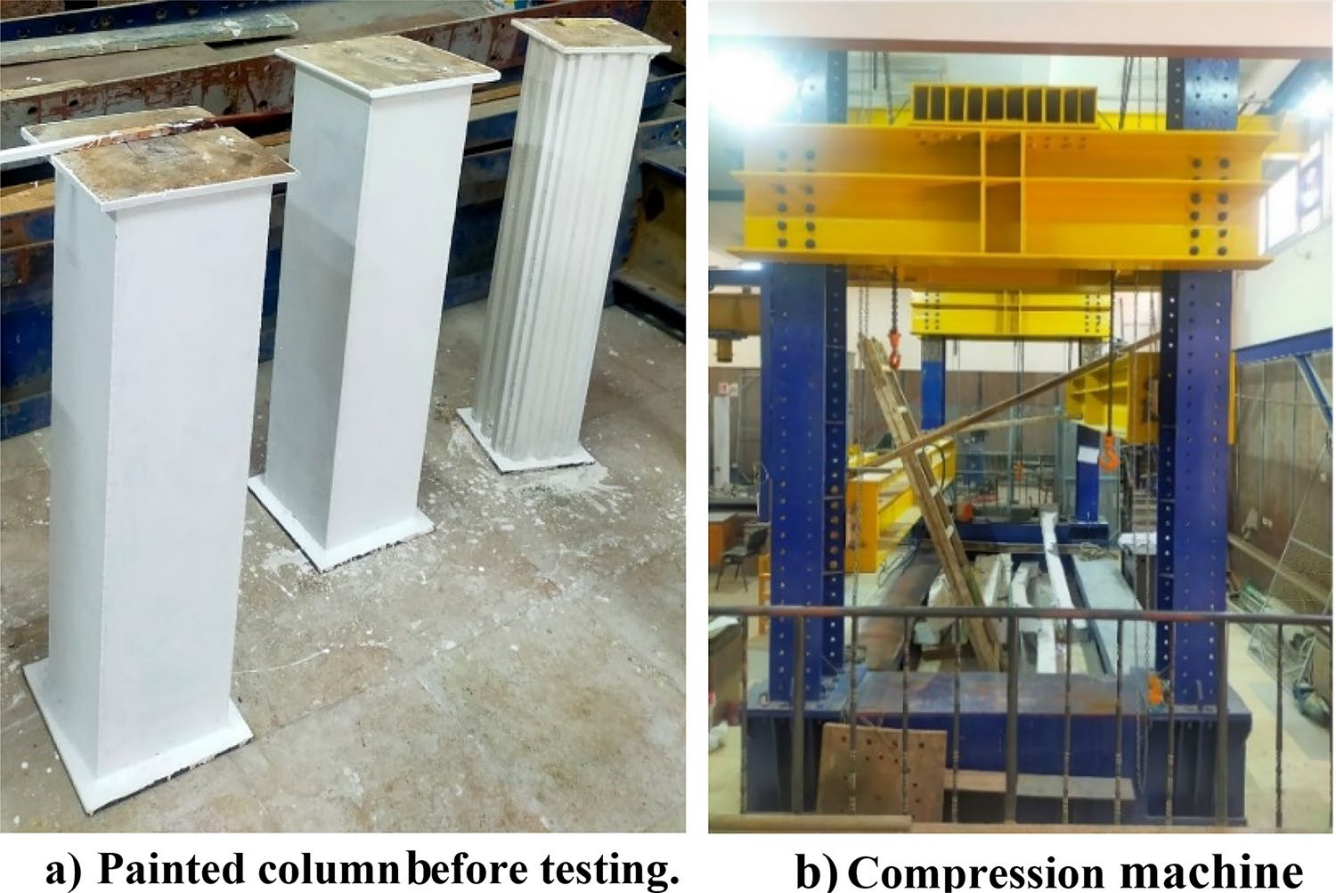
The specimens after preparation.

**Fig. 7:**
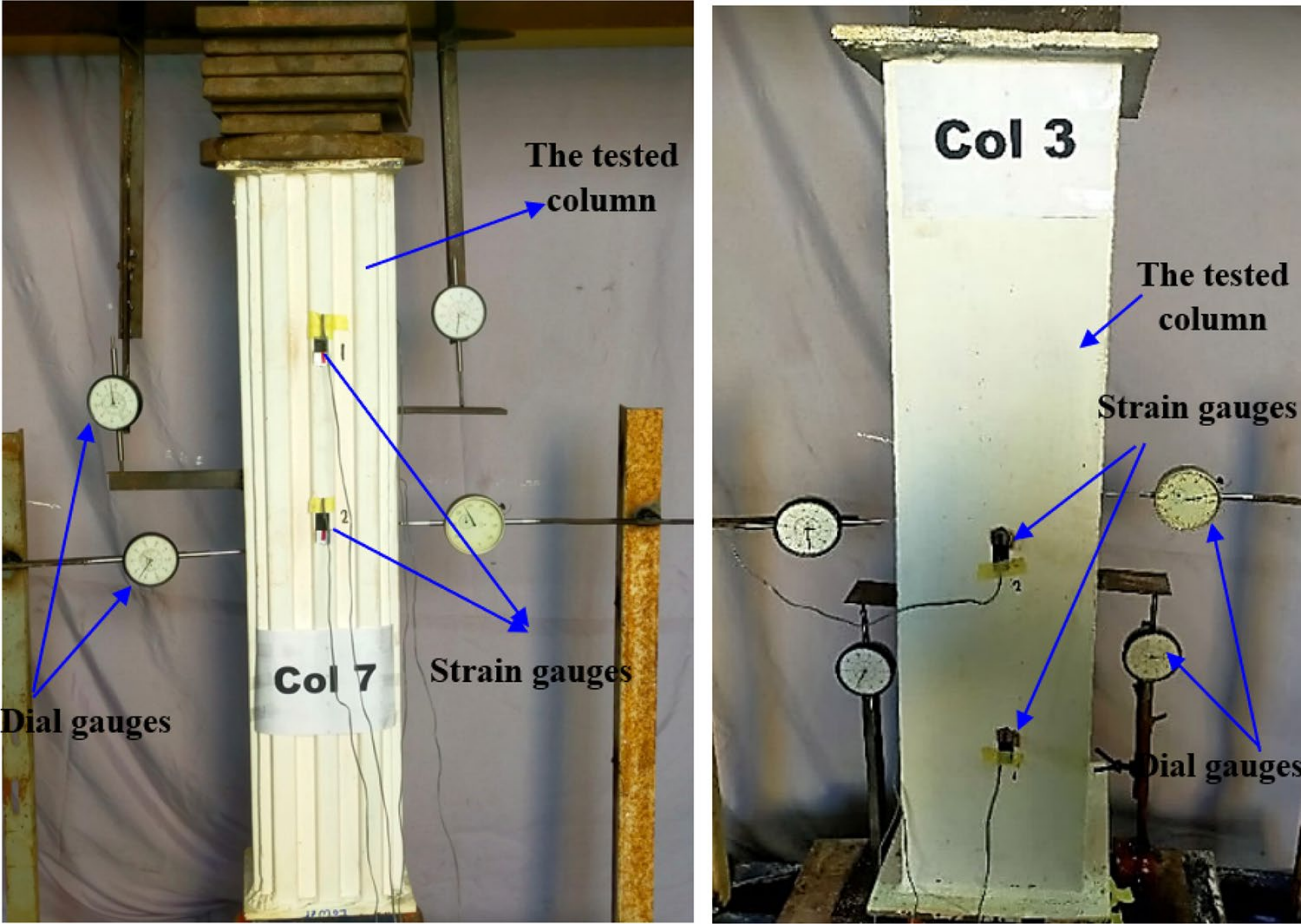
Test Set up and measurements.

All specimens were painted white to improve visibility, as shown in Fig. 6a. The test setup employed a uniaxial compression configuration, outlined in Fig. 6b. A concentrated load was applied to the specimens using a spreader plate. The experimental setup and arrangements are detailed in Fig. 7, where two dial gauges measured the axial shortening of the columns. The average dial gauge readings were used to determine the axial shortening for each specimen. Additionally, each specimen was equipped with four strain gauges, as depicted in Fig. 7. To ensure an even distribution of axial load in both the concrete core and steel tubes, a more rigid spreader plate replaced the specimens. A 3000 kN capacity hydraulic testing machine was employed to apply compressive axial force to the test specimens.

### Framework of machine learning techniques

#### ANN Model

**Fig. 8:**
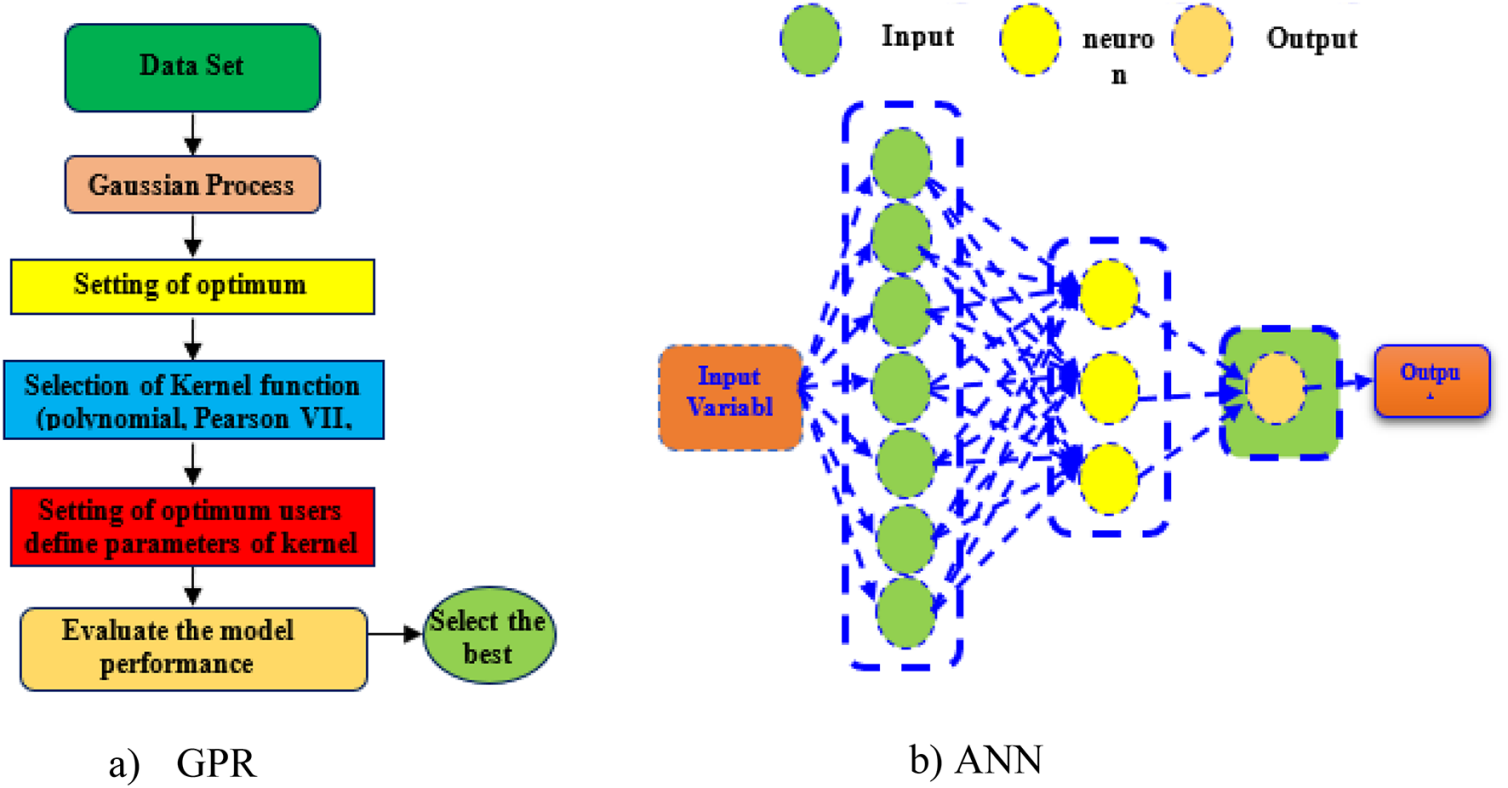
Schematic diagram of the proposed models.

The concept of ANN approach back to early attempts to simulate the sensory processing mechanisms of the brain^29, 30^. Typically, an ANN model comprises three main components: an input layer composed of neurons, one or more hidden layers of neurons, and an output layer. These layers are interconnected by weight vectors. The functioning of an ANN involves receiving environmental inputs through the input layer, which are then transmitted to the neurons in the hidden layer without immediate computation. Neurons within the hidden layer process this information, extracting pertinent features to establish the mapping from the input space to the output space. Ultimately, neurons in the output layer make predictions based on the information propagated from the preceding layers^29^. Figure 8a illustrates a standard architecture of an ANN model. Initially, each neuron computes the linear combination of outputs from the preceding layer and incorporates a bias into the resultant output. The weights, which determine the coefficient of the linear combination along with the biases, are established. Subsequently, the information undergoes processing within the neurons of the hidden layer utilizing a non-linear function, such as the sigmoid function. During the training process, convergence is gauged by ensuring that the RMSE derived from the training data is minimized or meets predefined criteria^31^.

#### GPR model

GPR model is a probabilistic and non-parametric technique in supervised learning, adept at capturing the underlying nonlinear and complex function mappings within datasets. It is rooted in the assumptions laid out by Rasmussen and Williams^32, 33^, suggesting that neighboring observations should convey information about one another, thereby providing a means to describe a prior directly over the space of functions. In this model, the mean and covariance of a Gaussian distribution (GD) are represented by vectors and matrices, respectively, with the GD serving as an overarching function. GPR delineates a prediction distribution akin to the test input, demonstrating the ability to discern complex patterns in data. Fundamentally, GPR embodies a collection of random variables characterized by a joint multivariate GD for any finite number. Let M×N denote the input and output domains, respectively, with n pairs (M_i_, N_i_) distributed independently and identically. In regression scenarios, the kernel function plays a crucial role in the GPR design process. Several kernels have been discussed in the literature^32, 34^. In this study, the polynomial (Poly) function of the GPR method is employed.

4 $$K(M,N){\text{ }}={\text{ }}{({\text{1}}+(K,M))^d},$$

where and are input variables, and is the degree of the polynomial kernel. This kernel function computes the internal product of input vectors and , adds 1 to it, and raises the sum to the power of . This kernel is particularly suitable for capturing nonlinear relationships between input variables in the GPR model. Additionally, Fig. 8b illustrates the schematic diagram of the GPR algorithm used to estimate the early-age CS.

#### Construction of ML models

To develop an accurate model for predicting the strength of CFDST columns, a comprehensive experimental database comprising 843 tests on CFDST columns subjected to axial loading was compiled (as detailed in the supplementary file). Although these experimental tests vary in their specific conditions, the dataset is extensive, diverse in sources, and representative of various real-world manufacturing scenarios^21, 24, 35–41^. The dataset focuses on CFDST columns subjected to axial loading, ensuring that the entire cross-sections, including concrete and steel pipes, are fully engaged under load. Only CFST columns incorporating normal concrete, high-strength concrete, and low-carbon steel pipes were included. Specimens with stainless steel pipes, aluminum pipes, recycled concrete, steel fiber concrete, and other non-standard materials were excluded to maintain consistency and relevance to the study’s objectives.

**Fig. 9:**
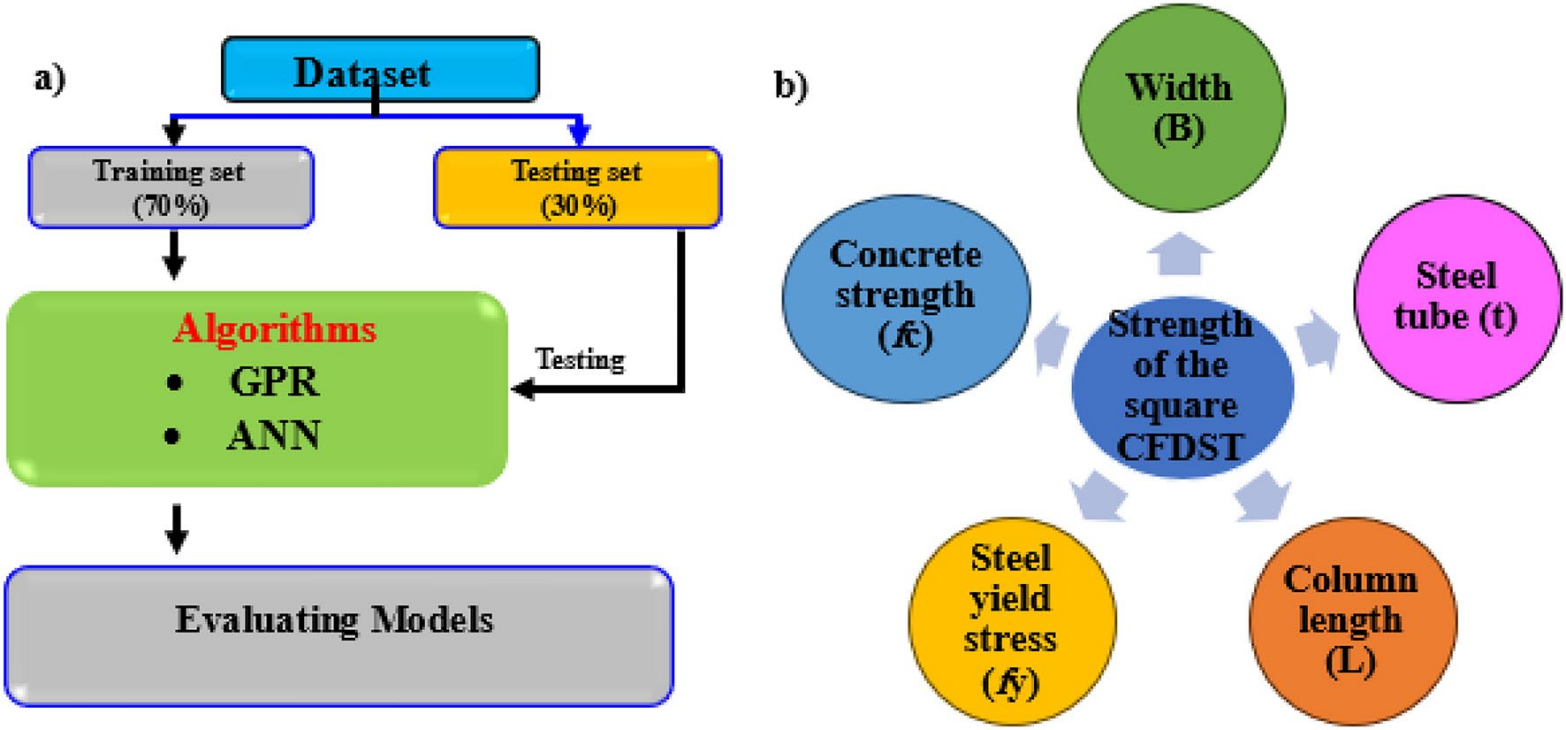
(**a**) The inputs of ML algorithms and (**b**) ML modeling framework in the current study.

The ML modelling framework, depicted in Fig. 9a, comprises three main steps: data gathering, algorithm implementation, and model validation and evaluation. Initially, data were collected based on selected input and output variables. Subsequently, the dataset was split into a training set and a testing set in a 70–30% ratio, respectively. The training set was used to build an effective ML model, while the testing set was employed to validate and evaluate the model’s performance^22, 35, 36^. In Figs. 5 and 9b features including the B, t, L, *f*y, and *f*c) were utilized as inputs to predict the strength of the square CFDST column as the output. Using an experimental dataset of 843 samples of square CFST columns, two ML models, ANN and GPR, were developed to predict the strength of the square CFDST column.

#### Performance measures

This study employs established statistical measures to evaluate the accuracy and reliability of both modeled and observed datasets, including conventional metrics like R^2^-value, RMSE, and MAE, alongside additional indices. These criteria are essential for assessing the predictive model’s accuracy and performance^35, 36^.

5 $$\:{R}^{2}=1-\frac{{\sum\:}_{i}{\left({y}_{i}-{\widehat{y}}_{i}\right)}^{2}}{{\sum\:}_{i}{\left({y}_{i}-{\stackrel{-}{y}}_{i}\right)}^{2}}$$

6 $$\:MAE=\frac{1}{n}\sum\:_{i=1}^{n}\left|{y}_{i}-{\widehat{y}}_{i}\right|$$

7 $$\:RMSE=\sqrt{\frac{1}{n}\sum\:_{i=1}^{n}{({y}_{i}-{\widehat{y}}_{i})}^{2}}$$

where y_i_, $\:{\widehat{y}}_{i}$and $\:{\stackrel{-}{y}}_{i}$ represent the actual, forecast, and average strength, respectively.

## Results and discussion

### Failure modes

**Fig. 10:**
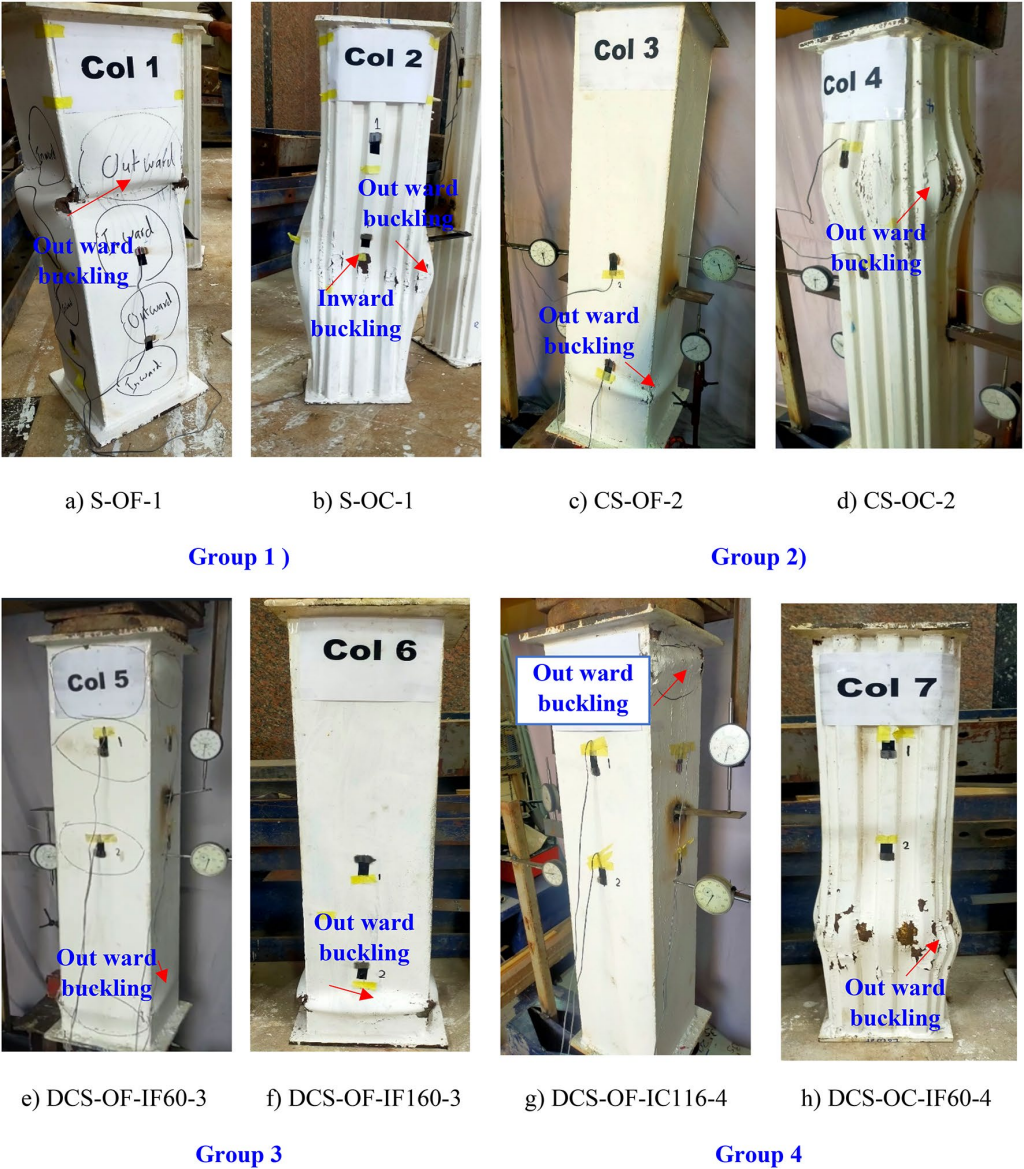
Mode of failure of external tube for CFDST columns.

**Fig. 11:**
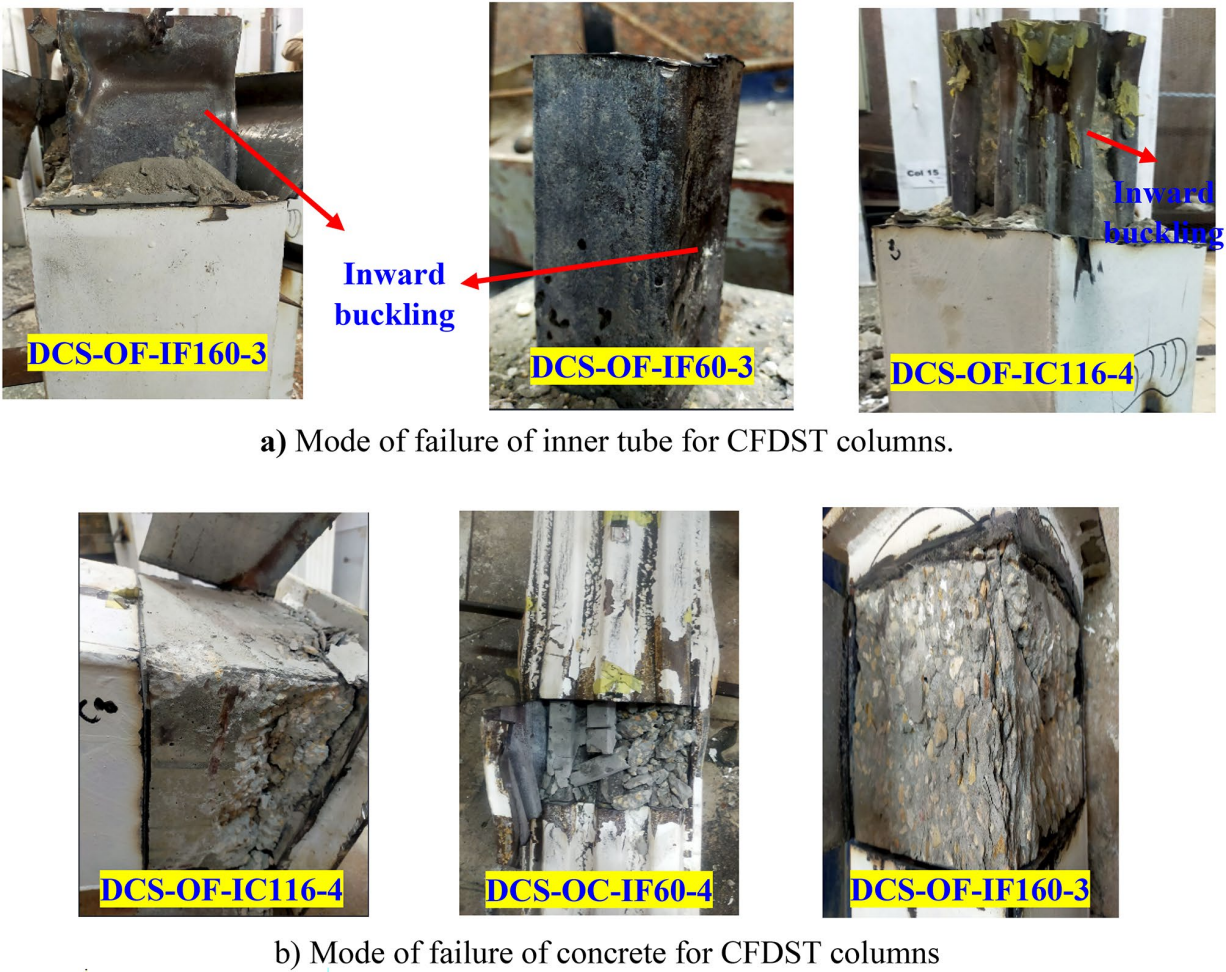
Failure Modes of CFDST columns.

The failure modes illustrated in Fig. 10 were observed upon completion of the tests, which terminated either upon visible damage or when the specimens’ ultimate bearing capacity dropped to 0.8 times ultimate strength. As depicted, while the specific failure modes varied, they generally involved external shell failures, such as fractures in external steel columns, local buckling of external flat steel tubes, or displacement of corrugated steel plates. Different configurations of internal steel tubes and concrete showed varying degrees of local buckling, as presented in Fig. 11. Specimens confined by corrugated steel plates exhibited less pronounced failure modes and local buckling extents. The *Group 1* in Fig. 10a and b exhibited a failure mode with inward and outward buckling in steel columns without filled concrete layers. Notably, in the traditional S-OF-1 (Fig. 10a), minor local buckling in the flat steel tube was observed as the axial load peaked, becoming more pronounced with increased axial displacement. In contrast, such local buckling was less evident in the S-OC-1 column (Fig. 10b). In *Group* 2 shown in Fig. 10c and d, the addition of filled concrete reduced the occurrence of crush failure modes. Up to reaching the peak load, no obvious failure mode was evident except for the corrugated steel plate becoming more compacted. As axial deformation increased, slight dislocation began to appear in the external corrugated plate, similar to what was observed in CS-OF-2 columns. On the other hand, for the case of composite columns, two main failure modes were observed with different external tube width to the internal tube width (*Group 3 and 4*), the concrete is crushed at a certain cross-section accompanied with local buckling of both tubes at the same cross-section, as can be seen in Fig. 10e to h. This is the same failure taking place in the CFST and CFDST columns with inward buckling at internal steel tube^38, 39^. However, the local buckling of the tubes is different from the case of the bare steel columns. Unlike that of the bare steel tubes, the local buckling of the external steel tubes in the composite columns took place in the outward direction, while that of the internal tubes is directed to the inside of the tubes as shown in Fig. 10e to h. However, the concrete and inner steel tube were crushed as shown in Fig. 11. Generally, it can be indicated that the proposed novel composite members (Fig. 10g) incorporating internal corrugated plates and external flat plates (DCS-OF-IC116-4) exhibit superior post-peak behavior and demonstrate better local stability compared to the other columns. This improvement is attributed to the inclusion of corrugated plates in the design.

### Load-displacement curves

**Fig. 12:**
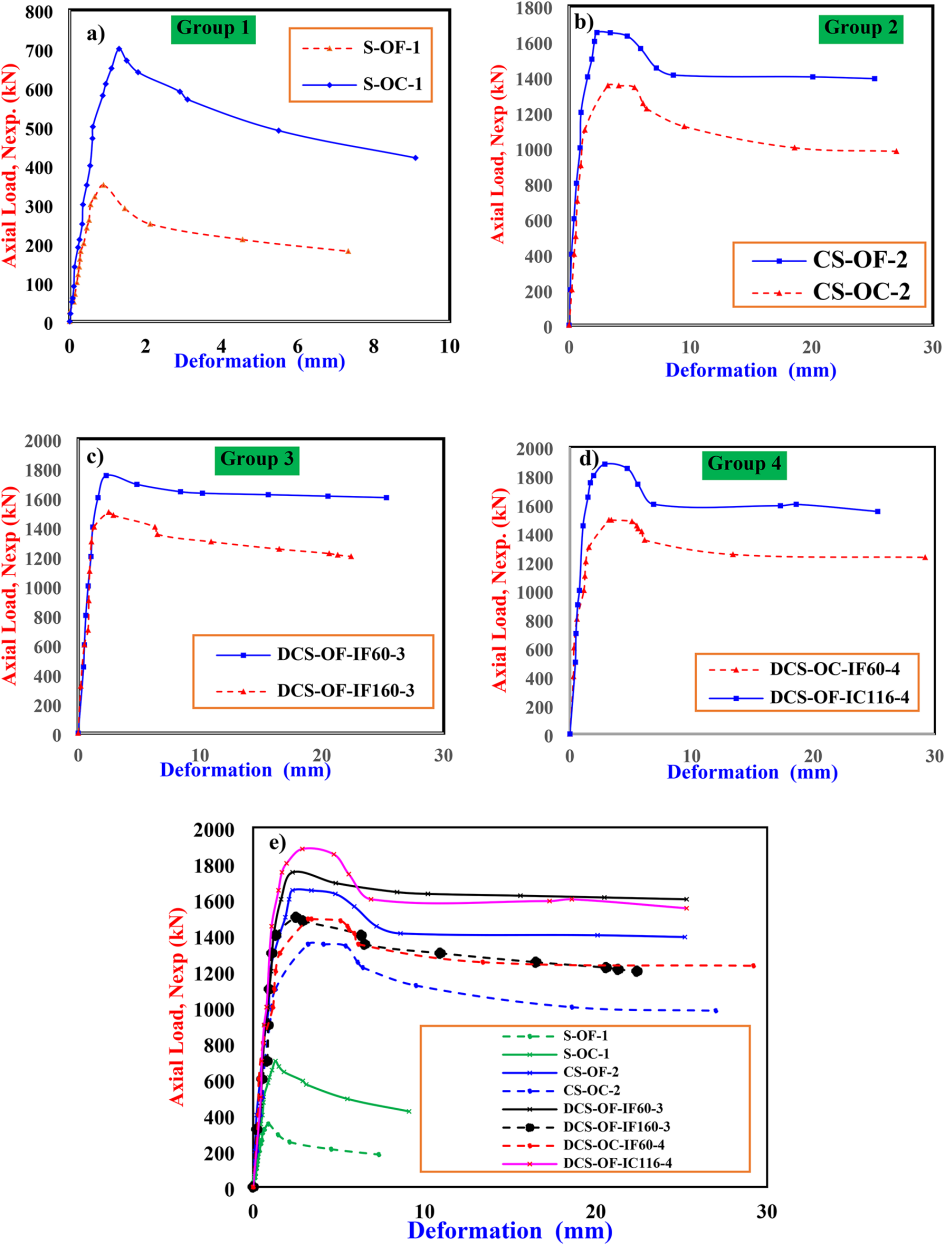
Load deformation behavior of CFDST specimens.

This study compares columns made of flat plates and corrugated plates with a constant area of steel. Figure 12 shows the axial load (kN) versus axial deformation (mm) responses for all samples, where the three identical specimens of each configuration exhibit nearly overlapping curves. For *Group 1*, the load-axial deformation responses of the bare specimens are provided in Fig. 12a. As can be observed, an increase in the strength of the corrugated column is about 2-times more than the results of the flat ones. On the other hand, the load-deflection response of the corrugated column shows similar response to that of the flat columns^21, 37, 38^. Upon comparing the area under each curve up to the peak point, it becomes evident that corrugation significantly enhances energy absorption and ductility. For Group 2, the load-axial deformation responses of the CS-OF-2 and CS-OC-2 columns, are provided in Fig. 12b. As can be observed, adding concrete to form the CS-OF-2 and CS-OC-2 columns provides a column with superior strength reached 1650 kN and 1350 kN, respectively. As shown in Fig. 12b, the stiffness of CS-OF-2 and CS-OC-2 columns is similar, while, there is an increase in strength of CS-OF-2 column is observed due to the increasing of area concrete than CS-OC-2 column. It worth noting that the area of concrete in CS-OC-2 column equal 0.63 of area concrete in CS-OF-2 columns. However, it is found that the strength in CS-OC-2 column is decreased by 0.85% compared CS-OF-2 column. Meanwhile, an increase in the strength of the CS-OF-2 of about 1300 kN results from forming the composite flat column compared with the bare ones and increase about 350 kN of the composite corrugated columns compared with the bare steel column S-OC-1. On other words, the load-deflection response of the composite column shows similar response to that of the bare columns until the ultimate load of the columns, from which the post-ultimate load stage become different. While the post-ultimate load stage in the bare steel columns decreases significantly, the composite column shows a stable descending branch with less degradation. This provides, in average, a strength index (SI) and ductility index (DI), calculated from Eqs. (8 to 10), of 92%. Additionally, the increase in stiffness, calculated in similar the same order of Eqs. (8 to 10), is about 55% for the corrugated composite columns.

8 $$\:SI=\frac{{p}_{u}}{{P}_{ue}}$$

9 $$\:{P}_{uo}={A}_{se}{f}_{ye}+{A}_{si}{f}_{yi}+{A}_{c}{f}_{ck}$$

10 $$\:DI=\frac{{\varDelta\:}_{u0.85}}{{\varDelta\:}_{y}}$$

Here, P_ue_ represents the individual bearing capacity of each material without considering their combined action; A_ce_, A_se_, and A_si_ denote the nominal cross-sectional areas of the sandwiched concrete, the external steel tubes, and the internal steel tubes, respectively. *f*_ck_ denotes the average compressive strength of concrete, where, *f*_ck_ = 0.67f_cu_. In addition to Δ_0.85_ is the axial displacement corresponding to the axial load reduced to 85% of ultimate loads and Δy is the yield displacement. For Group 3, the axial load-displacement curves of DCS-OF-IF60-3 and DCS-OF-IF160-3 columns with varying the width of internal steel tube with (B_i_= 60 mm and 160 mm, respectively) are provided in Fig. 12c. It is observed that the DCS-OF-IF60-3 and DCS-OF-IF160-3 columns had high strength and stiffness compared the composite column with single skin. By comparing the CFDST columns (Fig. 12c), the area of external steel tube is constant with different width of internal tube. It can be seen that the ultimate axial load decreased with the increase of the internal width^28, 42^, which reached 1750 kN and 1500 kN, respectively. Additionally, the narrow width exhibited the capacity of the column compared to the CFST columns.

For Group 4, the axial load-displacement curves of DCS-OF-IC116-4 and DCS-OC-IF60-4 column are provided in Fig. 12d. It is noticed by comparing DCS-OF-IC116-4 column, DCS-OC-IF60-4 which have the same area of external and internal steel skin. It is found that DCS-OF-IC116-4 sample has high compressive strength (1880 kN) more than column DCS-OC-IF60-4 (1490 kN) due to the increase of area concrete to 2-times compared to column DCS-OC-IF60-4. However, column DCS-OC-IF60-4 show high ductility more than column DCS-OF-IC116-4. Finally, Fig. 12e demonstrate that the ultimate axial force of DCS-OF-IF60-3 column is closely of DCS-OF-IC116-4 column, while the area of internal steel tube of DCS-OF-IC116-4 is more than area internal steel of DCS-OF-IF60-3 by 2.8 times approximately. The results of the experiments demonstrate that DCS-OC-IF60-4 column has greatest ductility and strength compared to other columns. Figure 12e show the comparison between all prepared columns. The results of load-displacement curve explore that the double-skin columns with internal corrugated tubes have high compressive strength and ductility more than the others column. However, the external corrugated steel columns show less strength compared to flat ones due to less confinement of concrete, and the decrease of area concrete for the corrugation shape. However, the external corrugated steel skin shows a good result for high (χ) ratio with less area of steel. Table 5 indicates that the increasing χ ratio is directly proportional to the A_s_/A_c_ ratio. It is evident that as the χ ratio decreases, the strength increases by 85% in composite flat columns. Conversely, when comparing the corrugated composite columns, column DCS-OC-IF60-4 shows lower compressive strength due to the concrete area in the external skin. However, column DCS-OC-IF60-4, which has corrugated plates in the internal skin and maintains a high χ ratio while keeping the steel area constant, demonstrates superior compressive strength. Specifically, it achieves a strength 1.25 times greater than that of a comparable composite flat column with the same external steel area.

**Table 5:** Properties of the CFDST columns specimens.

Column	${P}_{EXP}$ [kN]	A_se_ [mm^2^]	${A}_{si}$ [mm^2^]	${A}_{c}$ [mm^2^]	$\:{A}_{s,tot}/{A}_{c}$	$\:{B}_{i}/{B}_{e}$	SI	DI
DCS-OF-IF60-3	1750	2800	684	52,569	0.07	0.25	1.05	0.71
DCS-OF-IF160-3	1500		1884	30,569	0.16	0.66	1.03	0.62
DCS-OF-IC116-4	1490		684	34834.7	0.10	0.34	1.84	1.12
DCS-OC-IF60-4	1880		1884	42703.7	0.11	0.48	1.11	1.05

### Load versus longitudinal strain in CFDST columns

**Fig. 13:**
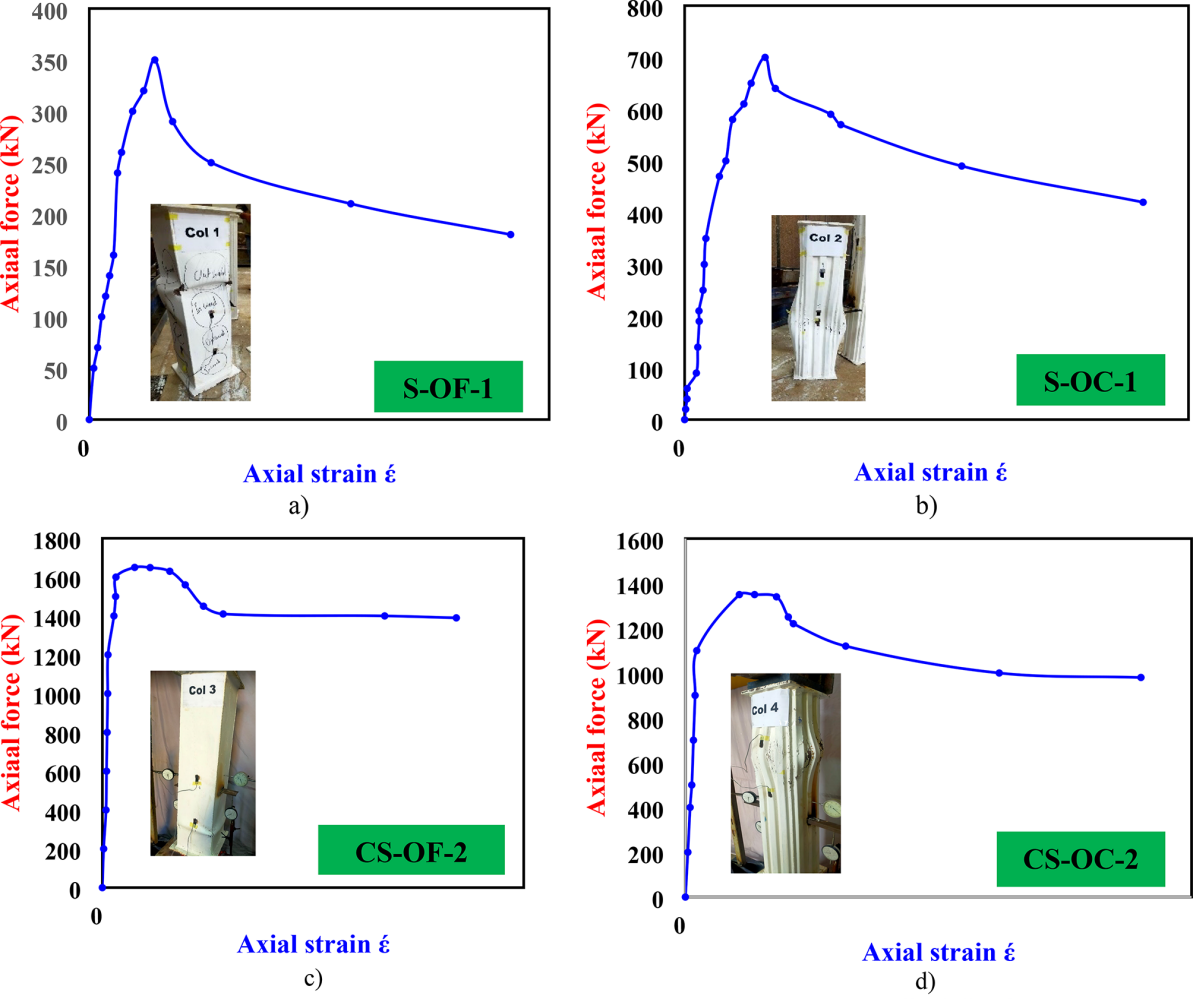

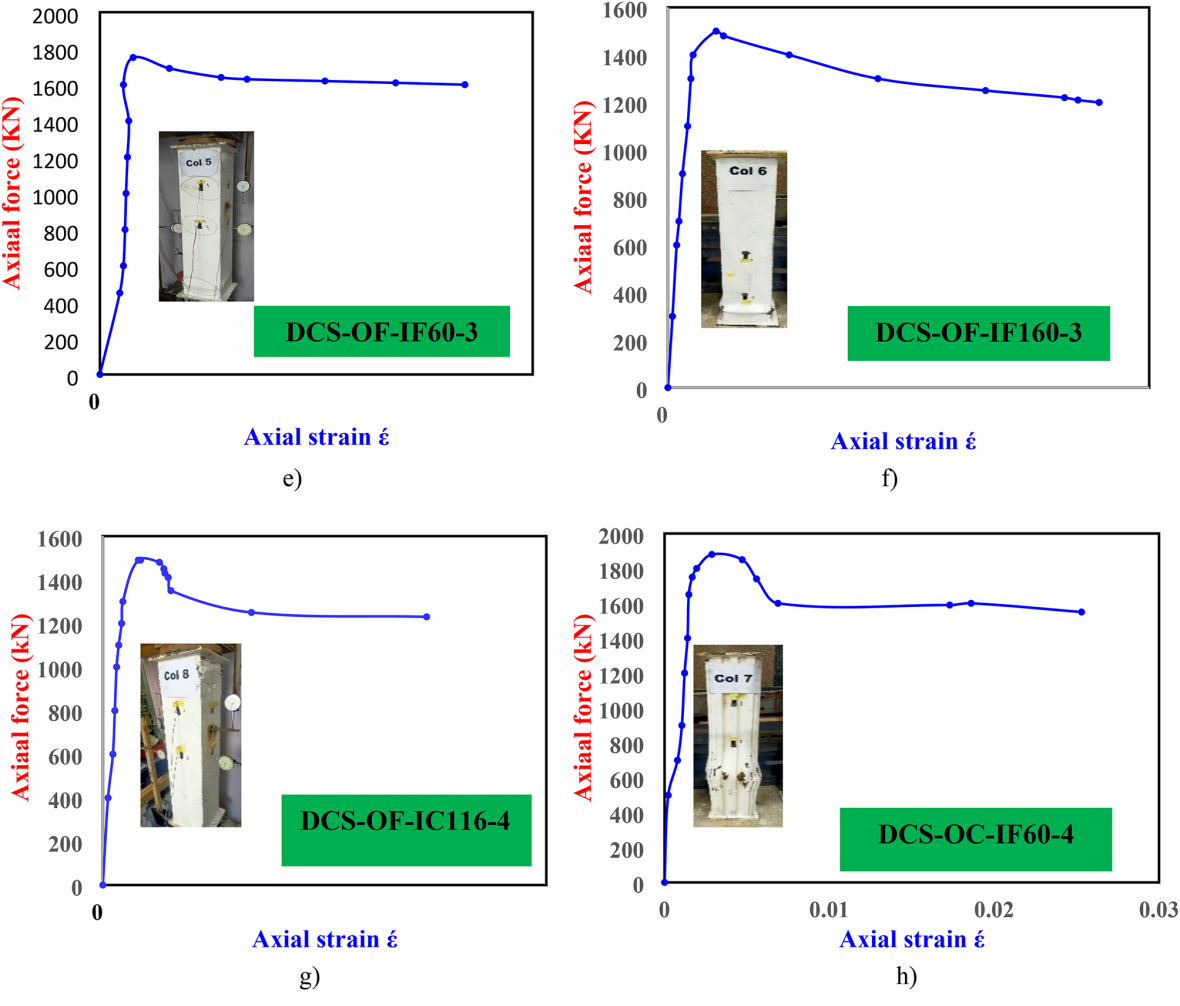
Axial force versus axial strain for prepared columns.

Figure 13(a–h) show load correlations versus longitudinal strain for various components of CFDST columns: external steel tube, internal steel tube, and sandwich concrete (points 1 in Fig. 6). It is evident that the internal steel tube has minimal impact on the load-bearing capacity compared to the sandwich concrete, which predominantly carries the load. The influence of concrete grade on the P-ε curves and ultimate strengths of these columns is also illustrated. This section explores the roles of the external steel tube, internal steel tube, and sandwich concrete in CFDST columns, emphasizing how their effects depend largely on steel grade and the confinement provided to the infilled concrete. The ultimate axial strength of the column significantly depends on factors like corrugated plate and internal tube dimensions^5^, combining the strengths of these cross-section components. Figure 13(a and b) depict responses of bare steel columns with flat and corrugated plates, showing an increase in the compressive strength of the external steel tube from 300 kN to 700 kN with similar strain values.

Figure 13(c and d) show responses of steel columns filled with concrete, comparing configurations reinforced with flat and corrugated plates. The flat plate configuration (CS-OF-2 column) saw a significant increase in compressive strength by 371.1%, while the corrugated plate (CS-OC-2 column) configuration improved by 285.7%, compared to the S-OF-1 column. Additionally, the corrugated CS-OC-2 column had maximum strain value which increased slightly from 0.008 to 0.027. Figure 13(e and f) and 13(g and h) illustrate responses of CFDST columns, specifically examining the impact of different widths of internal tubes on strain values and overall axial load strength. Among these configurations, DCS-OC-IF60-4 achieves the highest compressive strength, with strain values ranging from 0.008 to 0.0252. Overall, the ultimate strength of CFDST columns is primarily influenced by the type of external steel tube (flat or corrugated) and the width of the internal tube. The P-ε curves in Fig. 13(a-h) confirm that sandwich concrete in CFDST columns bears the majority of the load, typically accounting for approximately 49–54% of the total strength capacity. In comparison, external corrugated steel tubes and narrower internal tube widths contribute around 23–32% and 6–13% of the total strength capacity, respectively. These findings highlight the critical roles played by these components in determining the overall performance and load-bearing capacity of CFDST columns.

### Comparison between different codes

In this section, a comparison is made between the ultimate axial strengths of the tested CFDST columns with the strengths calculated by EC4, the ACI-318, the AISC-360, the design formula presented by Eurocode-4, ACI-318, AISC-360, GB 50,936 − 2014, AIJ (1997) and CECS 159:2004 and the equation proposed by Ding et al.^23^ and proposed equation by Hasan et al.^24^. The aim of comparisons is to find the best load predictor among these design models.

#### EC4 code

11 $$\:{P}_{EC4}=\left\{\begin{array}{c}{A}_{so}{f}_{yo}+{A}_{c}{f}_{c}+{A}_{si}{f}_{yi}\:\:\:\:\:\:\:\:\:\:\:\:\:\:\:\:\:\:\stackrel{-}{\lambda\:}>0.5\\\:{{\eta\:}_{a}A}_{so}{f}_{yo}+{A}_{c}{f}_{co}+{{\eta\:}_{a}A}_{si}{f}_{yi}\:\:\:\:\:\:\stackrel{-}{\lambda\:}\le\:0.5\end{array}\right.\:\:\:$$

Where, $\:{\eta\:}_{a}=0.25+(3+2\stackrel{-}{\lambda\:})\le\:1$ , A_so_, A_c_ and A_si_ represent the cross-sectional areas of the external tube, the concrete core and internal tube respectively.

#### ACI-318 code

12 $$\:{P}_{ACI}={A}_{so}{f}_{yo}+0.85{A}_{c}{f}_{c}+{A}_{si}{f}_{yi}$$

Where A_so_, A_c_ and A_si_ represent the cross-sectional areas of the external tube, the concrete core and internal tube respectively.* f*_y_ is the yield strength of the steel tube taken herein as the 0.2% proof stress (*σ*_0.2_).

#### AISC-360 code

13 $$\:{P}_{AISC}={A}_{so}{f}_{yo}+{0.95A}_{c}{f}_{c}+{A}_{si}{f}_{yi}$$

Where A_so_, A_c_ and A_si_ represent the cross-sectional areas of the external tube, the concrete core and internal tube respectively.

#### Ding et al.^23^ equation

14 $$\:{P}_{Ding\:et.al}={KA}_{so}{f}_{yo}+{A}_{c}{f}_{c}+{A}_{si}{f}_{yi}$$

Where,$$\:\:K=1.2+0.15{X}_{O}-0.3\sqrt{{X}_{0}}\:\:,0\le\:{X}_{o}\le\:0.77$$, X_o_=b_i_^2^/b_e_^2^,

A_so_, A_c_ and A_si_ represent the cross-sectional areas of the external tube, the concrete core and internal tube respectively.

#### GB 50,936 − 2014

The code GB 50936–2014 classifies a CFST consisting of two distinct materials (steel and concrete) as a novel material. In axial compression, the CFST is expressed as follows:

15 $$\:{P}_{GB\:50936-2014\:}=\phi\:\:{N}_{O}$$

Where, $\:{N}_{o}$ is the design value of axial compression strength bearing capacity of CFST column, which calculated by,

16 $$\:{N}_{o}=\:{f}_{sc}\:{A}_{sc}$$

17 $$\:{f}_{sc}=\left(1.212+\theta\:{B}_{S}+\:{\theta\:}^{2}{C}_{c}\right){f}_{c}$$

18 $$\:\theta\:={\propto\:}_{sc}\:\left({f}_{yo}+{f}_{yi}\right)/{f}_{c}$$

19 $$\:{\propto\:}_{sc}={A}_{st}\:/{A}_{C}$$

20 $$\:{B}_{S}=(0.131\times\:\left(\frac{{f}_{yo}+{f}_{yi}}{213}\right)+0.723$$

21 $$\:{C}_{C}=-\left(0.070\times\:\frac{{f}_{c}^{\prime}}{14.4}\right)+0.026$$

22 $$\:\phi\:=\frac{1}{2{\stackrel{-}{Y}}_{sc}}\left[{\stackrel{-}{Y}}_{sc}^{2}+\left(1+0.25{\stackrel{-}{Y}}_{sc}\right)-\left(\sqrt{\left({\stackrel{-}{Y}}_{sc}^{2}+\left(1+0.25{\stackrel{-}{Y}}_{sc}\right)\right){)}^{2}-4{\stackrel{-}{Y}}_{sc}^{2}}\right)\right]$$

23 $$\:\stackrel{-}{{Y}_{sc}}=\frac{{\gamma\:}_{sc}}{\pi\:}\:\sqrt{\frac{{f}_{sc}}{{A}_{sc}}\:}\:\approx\:0.01{\gamma\:}_{sc}\left(0.01\right({f}_{yo}+{f}_{yi)}$$

#### AIJ (1997)

According to AIJ (1997), the following formula determines the ultimate axial bearing capacity of a square CFST column.

24 $$\:P_{{AIJ\:\left( {1997} \right)}} = \left( {F \times \:1.27A_{{st}} } \right) + ({}^\backprime f_{c} \times \:0.85A_{c} )$$

Where, the ultimate axial bearing capacity is P_AIJ (1997)_, $\:F=(0.7{f}_{su},{f}_{y})$, choose the minimum value, “F” is the accepted standard for steel strength and A_st_ is the area of outer and inner steel tube.

#### CECS 159:2004

The Chinese local code CECS 159:2004 determines the ultimate axial bearing capacity of the square CFST column by adding the strengths of the steel tube and concrete.

25 $$\:{P}_{CECS\:159:2004}=\phi\:\left\{\left({A}_{so}\times\:{f}_{yo}\right)+\left({A}_{si}\times\:{f}_{yi}\right)+({A}_{c}\times\:\stackrel{\prime }{{f}_{c})}\right\}$$

When $$\:{\gamma\:}_{o}\le\:0.215$$,

26 $$\:\phi\:=1-0.65{\gamma\:}_{o}$$

When $$\:{\gamma\:}_{o}>0.215$$,

27 $$\:\phi\:=\frac{1}{2{\gamma\:}_{o}^{2}}\left\{\left(0.965+0.300{\gamma\:}_{o}+{\gamma\:}_{o}^{2}\right)-\sqrt{(0.965+0.300{\gamma\:}_{o}+{\gamma\:}_{o}^{2}{)}^{2}-4{\gamma\:}_{o}^{2}}\right\}$$

28 $$\:{\gamma\:}_{o}=\frac{L}{{R}_{O}}$$

29 $$\:{R}_{o}=\sqrt{\frac{{I}_{so}+{I}_{si}+\frac{{I}_{c}{E}_{c}}{{E}_{s}}}{{A}_{st}+\frac{{A}_{c\:}{f}_{c}}{f}}}$$

Here, I_c_, I_s_, E_c_, E_s_ and f are respectively the steel and concrete moments of inertia, the modulus of elasticity of concrete and steel, and the design value of steel tensile strength.

#### Hasan et al.^24^ equation

axial compressive ultimate strength (P _Md Mehedi Hasan et al._) of cruciform, special-shaped CFST columns.

30 $$\:{P}_{\mathbf{M}\mathbf{d}\:\mathbf{M}\mathbf{e}\mathbf{h}\mathbf{e}\mathbf{d}\mathbf{i}\:\mathbf{H}\mathbf{a}\mathbf{s}\mathbf{a}\mathbf{n}\:\mathbf{e}\mathbf{t}.\:\mathbf{a}\mathbf{l}}={C}_{c}\left\{\left(F\times\:1.27({A}_{so}+{A}_{si}\right)+({\stackrel{\prime }{f}}_{c}\times\:0.85{A}_{c})\right\}$$

31 $$\:{C}_{c}=0.4853+(0.2792\times\:\xi\:)$$

**Fig. 14:**
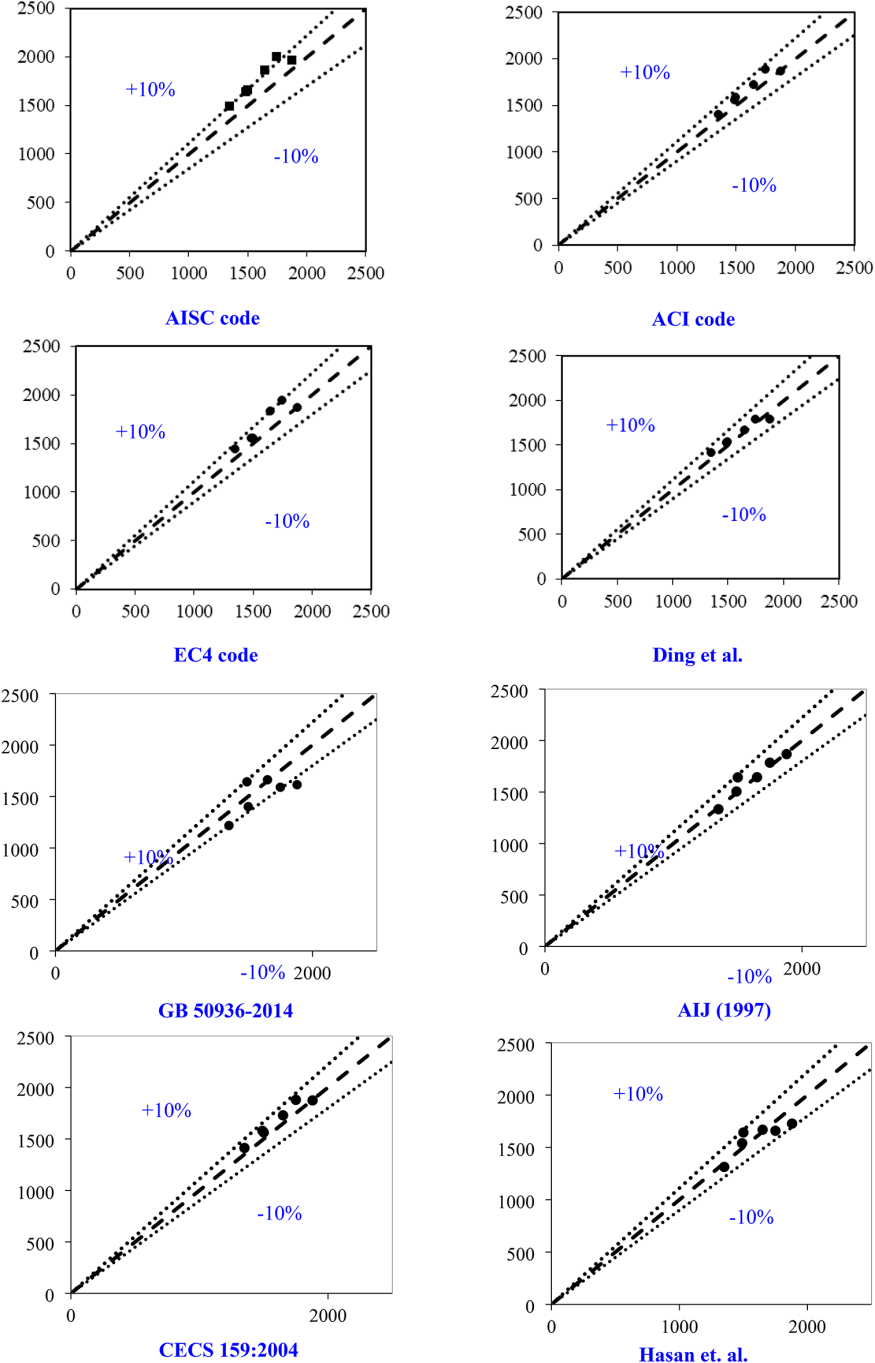
Comparison between different codes.

Table 6 presents the strengths calculated with the above design methods, from which it can be noticed that, in average, the following strength methods of the steel tubes is approximate to each other, with an average ratio of unity associated with the least standard deviation value of 0.03. On the other hand, a detailed comparison of the $\:{P}_{Exp}$ values for the CFDST columns with the original predictions from Eurocode 4, ACI code, AISC code, the design equation by Eurocode-4, ACI-318, AISC-360, GB 50,936 − 2014, AIJ (1997) and CECS 159:2004 and the equation proposed by Ding et al.^23^ and proposed equation by Hasan et al.^24^. is presented in Fig. 14, considering the calculations of the effective area. In these figures, a diagonal line is added which indicates that the design strength is typical to the experimental one. Two lines representing the boundaries of strengths with ± 10% compared with unity were also added, which might be acceptable in the design of the current composite columns. However, all design models involve strengths with more than ± 10% of the test strengths.

**Table 6:** Comparison between experimental study and different codes.

Column	$\:{P}_{Exp}$ [kN]	$\:\frac{{P}_{Exp}}{{P}_{GB50936-204}}$	$\:\frac{{P}_{Exp}}{{P}_{AIJ\:1997}}$	$\:\frac{{P}_{Exp}}{{P}_{CECS\:159:2004}}$	$\:\frac{{P}_{Exp}}{{P}_{Md\:Mehedi\:et.\:aling\:etal.}}$	$\:\frac{{P}_{Exp}}{{P}_{AISC}}$	$\:\frac{{P}_{Exp}}{{P}_{ACI}}$	$\:\frac{{P}_{Exp}}{{P}_{EC4}}$	$\:\frac{{P}_{Exp}}{{P}_{Ding\:etal.}}$
CS-OF-2	1650	0.992	1.00	0.95	0.98	0.89	0.96	0.90	0.99
CS-OC-2	1390	1.108	1.01	0.96	1.02	0.91	0.96	0.94	0.96
DCS-OF-IF60-3	1750	1.100	0.98	0.93	1.05	0.87	0.93	0.90	0.98
DCS-OF-IF160-3	1500	1.070	0.91	0.96	0.92	0.90	0.95	0.97	0.98
DCS-OF-IC116-4	1490	0.907	0.99	0.94	0 97	0.91	0.96	0.96	0.98
DCS-OC-IF60-4	1880	1.164	1.10	1.02	1.09	0.96	1.01	1.06	1.13
AVE	1.05		0.98	0.96	1.006	0.91	0.96	0.96	1.002
SD	0.08		0.03	0.02	0.05	0.03	0.03	0.055	0.057

### Prediction of square CFDST column strength

This research integrates ANN and GPR models using a comprehensive dataset of 843 dataset compiled from various experimental programs to predict the strength of square CFDST column. During training, approximately 70% of the dataset was randomly selected, while the remaining 20% was reserved for validation. Key parameters influencing the axial compressive capacity of CFDST columns (Nexp.) were identified, including B_e_, L, t_o_, *f*_y_, and *f*_c_^21, 35–39^. The ANN approach employed in this study featured an input layer with six neurons and an output layer with a single neuron using a linear activation function. The optimal number of neurons in the hidden layer was determined through iterative testing, ranging from one to ten neurons^35^. Following thorough evaluation, the configuration that demonstrated the best performance was selected for further model refinement. The final ANN setup for predicting compressive strength included a single hidden layer with four neurons^35, 38, 39^.

**Fig. 15:**
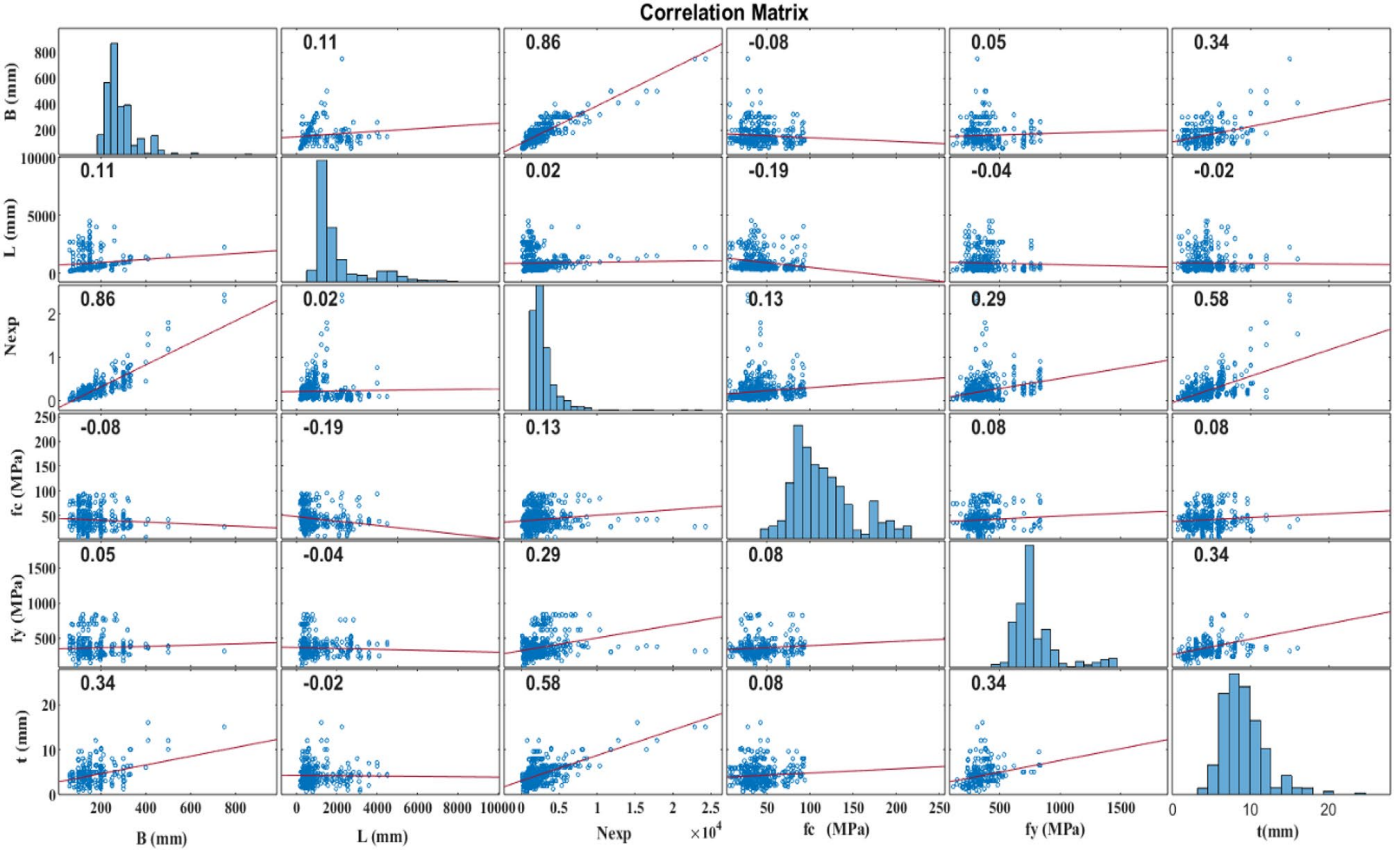
Correlation between input features and output (axial force).

**Fig. 16:**
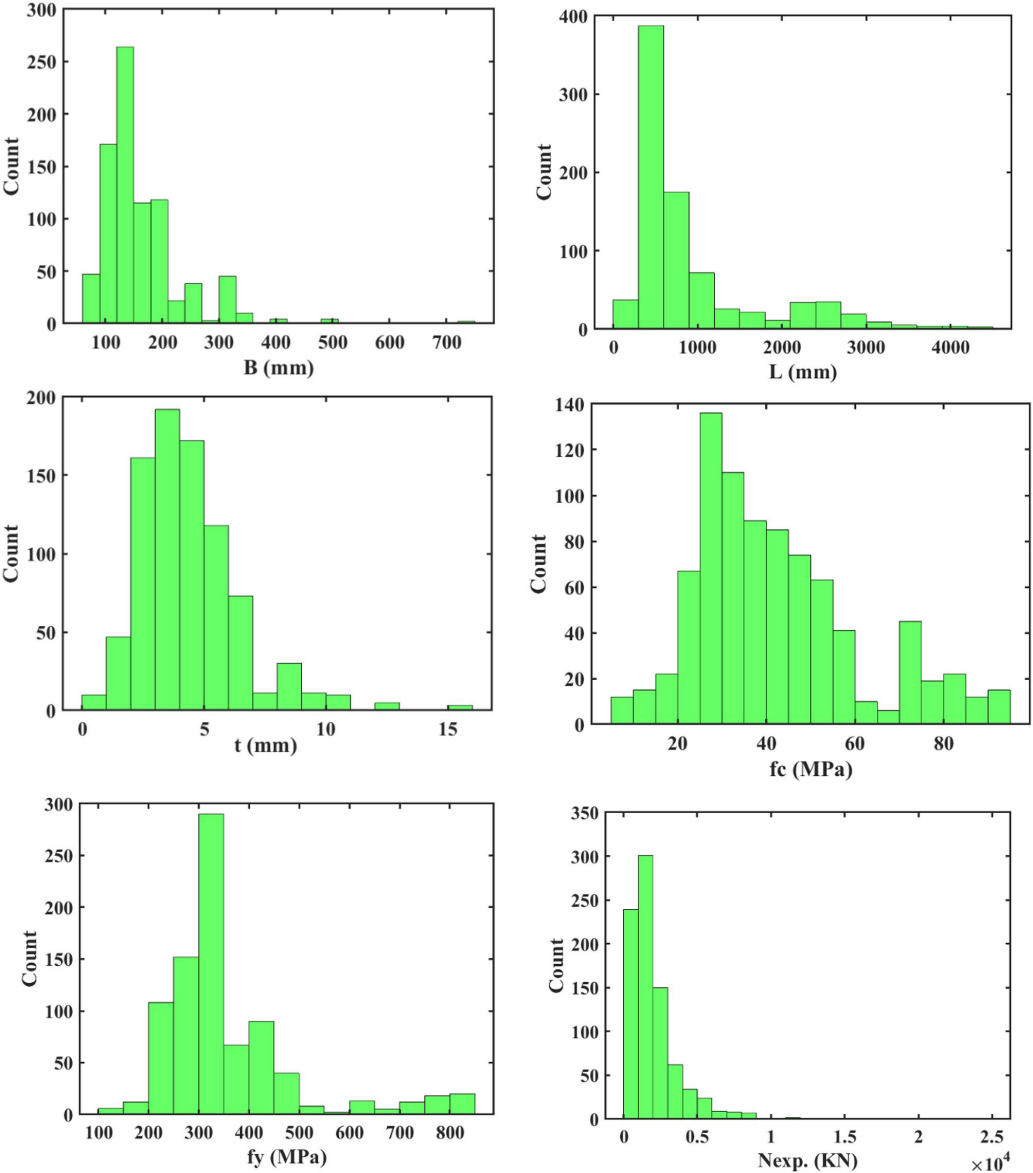
Distribution of the dataset (input features and output (axial force)).

**Fig. 17:**
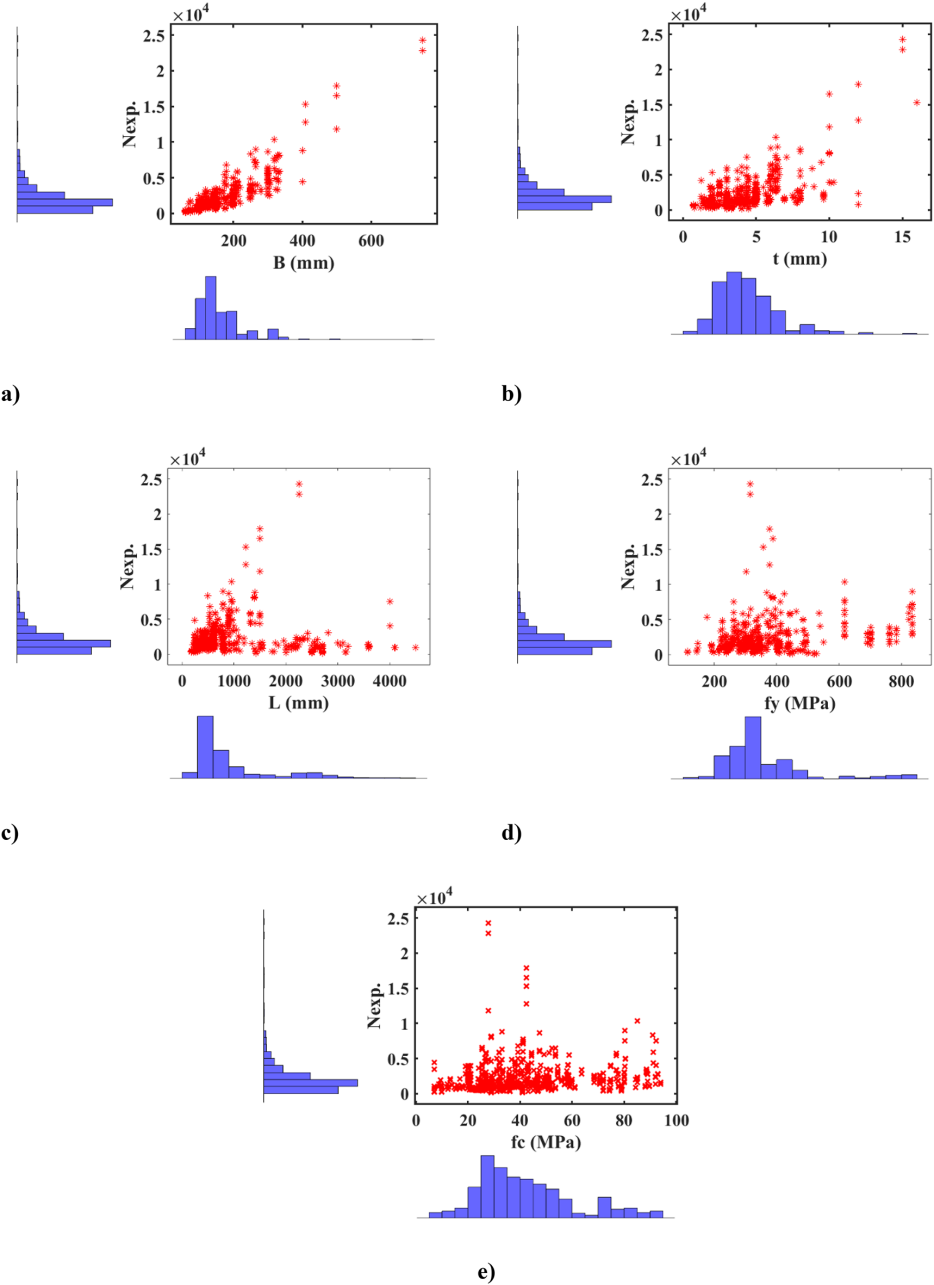
Relationship between input and output variables.

A comparative analysis assessed the performance of different trained models, identifying the model with the highest accuracy. To illustrate the relationships between independent and dependent variables, a correlation matrix was employed, which evaluates linear correlations between pairs of variables, often expressed through R^2^. Figure 15 displays these correlations along with distributions for each parameter. Overall, the relationships between variables are not particularly strong or significant. The highest correlation among input parameters is observed between B_e_ and t and between *f*y and t (mm), yielding an R^2^ of 0.34. Following this, the relationship between L and *f*c shows R^2^ of 0.19. Regarding input-output relationships, the variable B exhibits the highest R^2^ (0.86) in predicting N_exp_, followed by t with an R^2^ of 0.58. These findings suggest that developing a multi-input model with enhanced accuracy is crucial based on these initial regression analyses. As anticipated, the correlation matrix reveals the strongest correlations (0.86, 0.58) between the N_exp_ and the input attributes (B, t), as depicted in Fig. 15. To showcase a broad range of column parameters sourced from the database, distribution of the datasets for inputs and output are presented in Fig. 16. Additionally, the relation between the input and output variables are presented in Fig. 17. These observations highlight the variability in column parameters and demonstrate the importance of considering a wide spectrum of properties in developing robust design expressions.

### Model evaluation

**Fig. 18:**
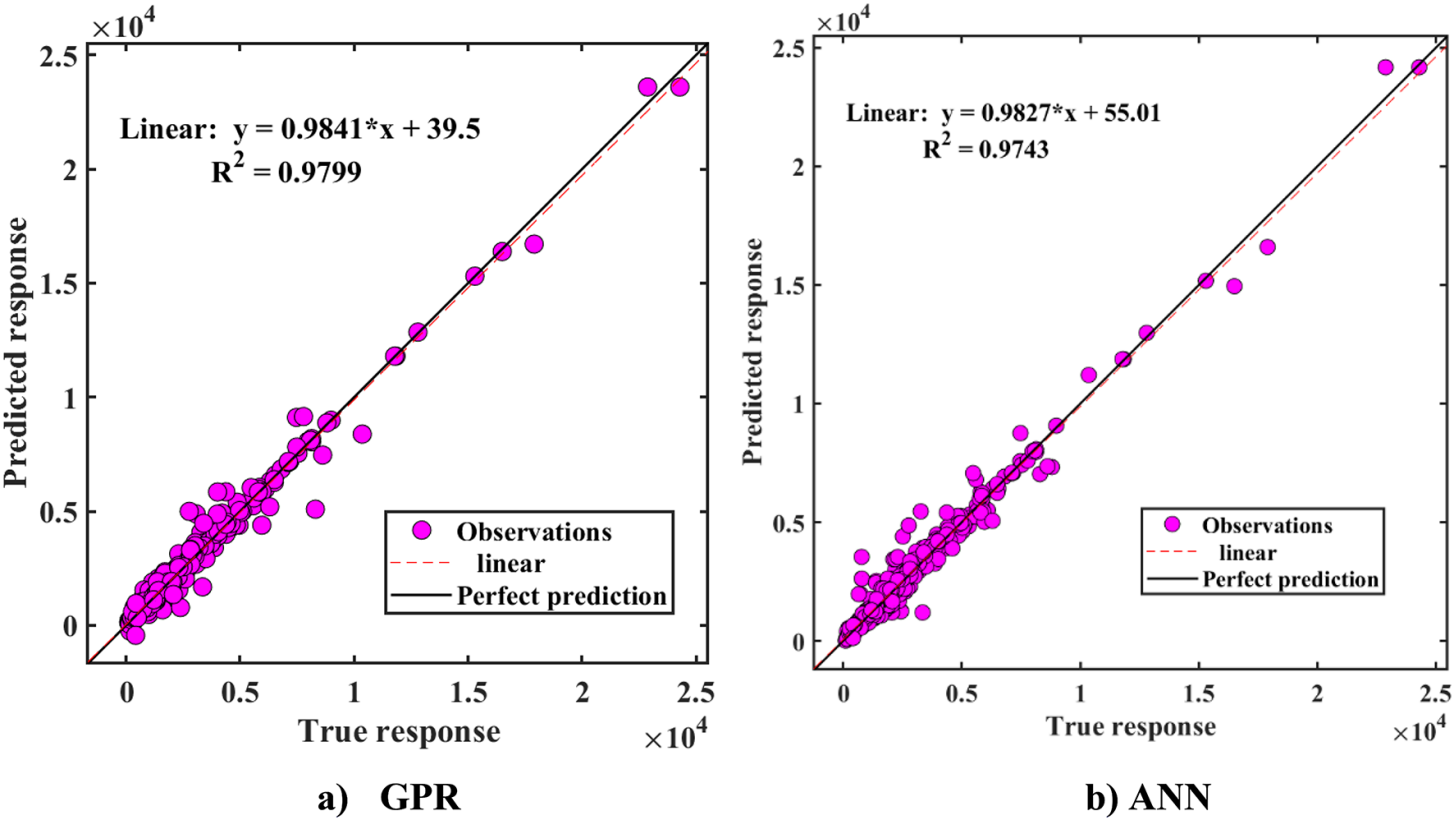
Actual vs. predicted N_exp_ using the ML algorithms.

Table 7 presents the indicators for the prediction of Nexp. during both the training and testing stages. The table indicates that the R-value for the GPR model is approximately 0.99 for both datasets, demonstrating that the proposed models are reliable and effective in predicting N_exp_. However, the RMSE and MAE values for the ANN model during the testing stage are 197.21 kN and 328.61 kN, respectively, which indicate that the ANN model performs less effectively compared to the GPR model (with RMSE = 267.80 kN and MAE = 157.11 kN). Figure 18 plots the regression lines comparing the N_exp_ predictions of ANN and GPR models against experimental values for datasets. It is evident from the figure that the slope of the regression line for training instances is 0.9841 for GPR and 0.9827 for ANN. Both slopes are close to the ideal fit of 1, indicating a strong alignment between predicted and experimental values for N_exp_ across both models.

**Table 7:** Indicators for the prediction of the proposed models.

		*R*	RMSE	MSE	MAE	MAPE
GPR	Training	0.99	267.80	71,717	157.11	10.5
	Testing	0.97	303.21	91,937	176.29	10.5
ANN	Training	0.98	352.80	1.244 × 10^5^	191.93	13.50
	Testing	0.97	328.61	1.079 × 10^5^	197.21	12.50

**Fig. 19:**
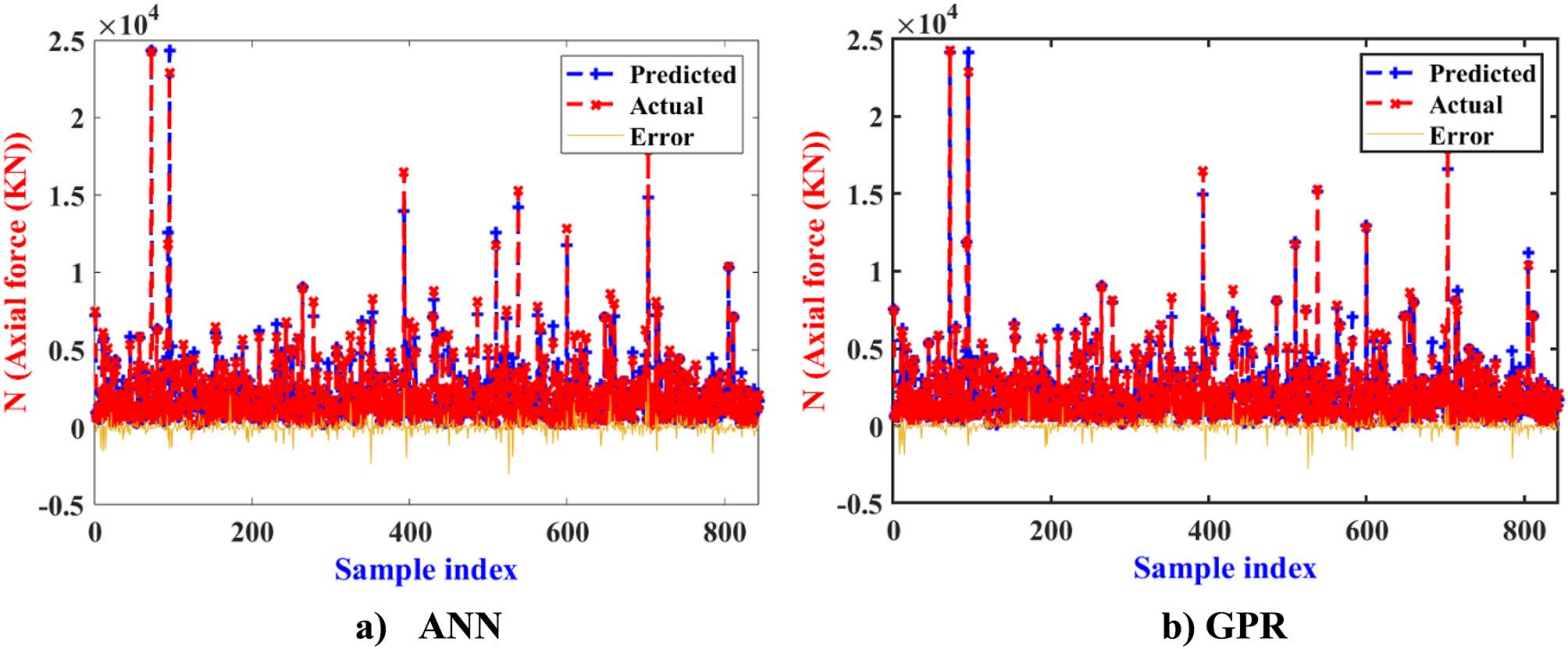
Actual, predicted N_exp_ and error values using ML algorithms.

Figure 19 displays the prediction results using both ML algorithms. The results indicate that for most samples, there is a relatively small distance between the experimental and predicted values, affirming the feasibility of constructing a strength prediction model for columns using ML algorithms. The absolute error calculations, represented in the orange histogram, reveal that the absolute errors of the majority of samples fall within the range of ± 0.5 * 10^5^ kN. Specifically, GPR algorithm demonstrates the best performance on the training set, achieving extremely low absolute errors compared to other algorithms. Moreover, the selected ML algorithms exhibit similar prediction performances on the testing set, indicating robustness and reliability across different datasets. These findings underscore the efficacy of ML techniques in accurately predicting N_exp_ for column designs, validating their application in practical engineering scenarios.

### SHAP interpretations

**Fig. 20:**
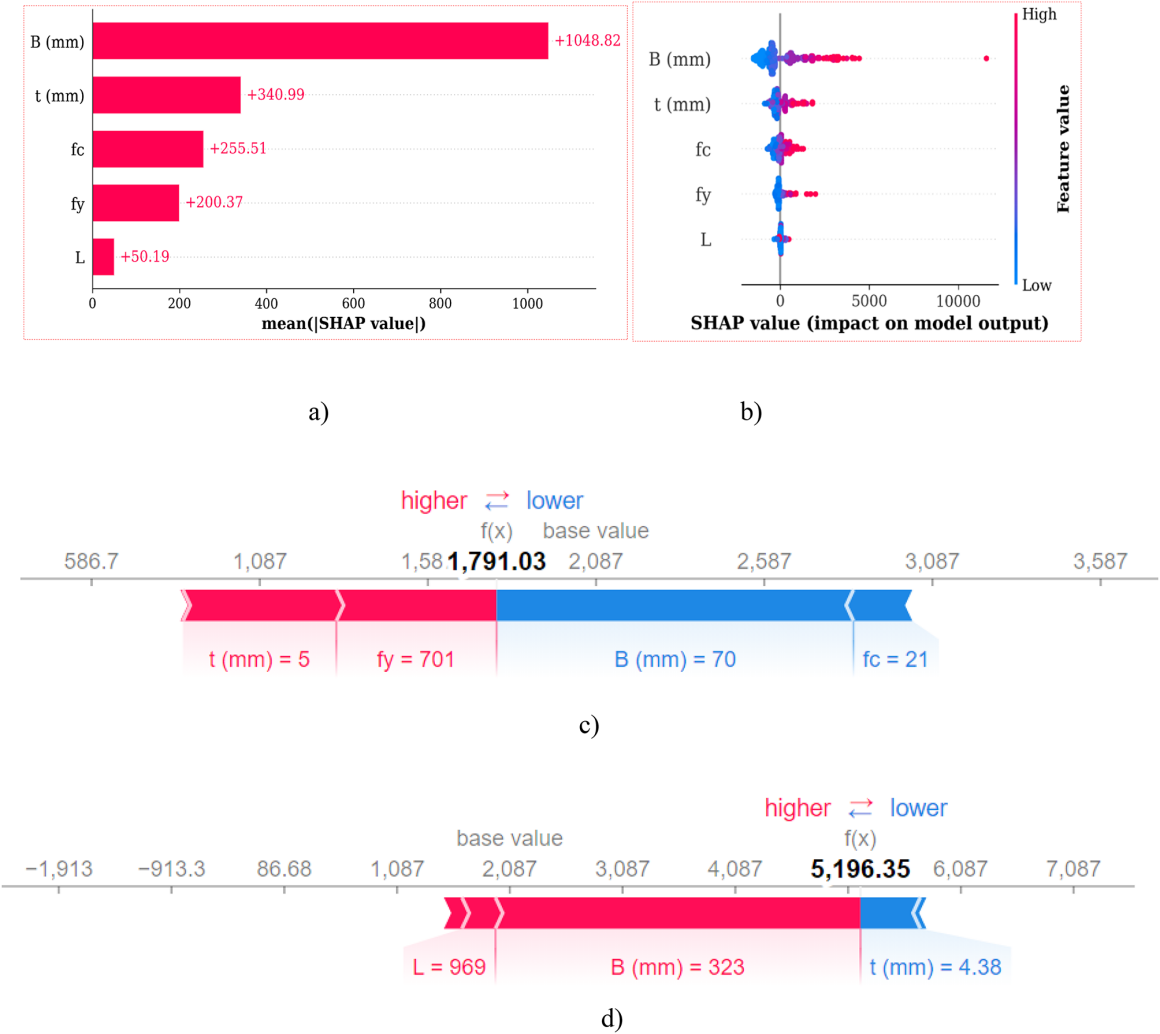
SHAP interpretations of GPR model (**a**) Mean absolute SHAP values (**b**) SHAP global explanation (**c**) and (**d**) SHAP force plot on selected instances for local interpretation.

Previous studies often overlooked the use of machine learning interpretability methods to explain prediction outcomes, necessitating post-hoc explanations as models became more complex. In response, SHAP (SHapley Additive exPlanations) was employed in this study to uncover the underlying logic behind predictions, indicate variable interactions with outcomes, and provide insights into specific instances. The GPR model demonstrated superior performance over the ANN model, prompting the application of SHAP to interpret the GPR model. Figure 20a and b depict the mean absolute SHAP values and global explanations of the GPR model, respectively. Feature values are represented visually with a color scheme where blue indicates lower values and red indicates higher values. In Fig. 20a, the B exhibited the highest mean SHAP value, indicating its significant impact on the model output. Conversely, in Fig. 20b, the thickness of the external plate emerged as the predominant factor. SHAP efficiently computed these mean values to reflect their influence on the model output. Additionally, SHAP analysis revealed minimal influence of column length on the output and emphasized that the *f*_c_ had a greater effect than steel f_y_. Figure 20b further illustrates how mean SHAP values correspond to original feature importance metrics, offering insights into the relative importance of each feature in the GPR model’s predictions.

Furthermore, SHAP explanations surpass the correlation matrix by quantifying the influence of each variable across its entire range, offering insights that simple correlation analysis might overlook. This comprehensive analysis enhances our understanding of how each feature contributes to the predictions made by the model. In addition to global interpretations, Fig. 20c and d present selected instances for local explanations, focusing on specific predictions obtained from SHAP force plots. For instance, in Fig. 20c, lower values of column width and concrete grade are associated with decreased N_exp_. The bolded value (base value + SHAP values) indicates the prediction generated during the ML training process, highlighting the unique contributions and interactions of variables at any given point. Moreover, Fig. 20d illustrates that column width positively impacts N_exp_, while column length also shows a positive influence, whereas column thickness has a negative effect. In summary, SHAP explanations provide detailed insights into how individual variables contribute to the model’s predictions, reinforcing understanding from both global and local perspectives.

## Summary and conclusions

This paper focuses on comparing columns constructed using flat plates versus corrugated plates, while maintaining a constant area of steel. The study explores various column types, including hollow tubes, CFST, and CFDST columns. Additionally, it examines effect of different parameters on this type of columns. The following conclusions can be drawn from this study:

1. The study demonstrates that corrugated tubes significantly enhance the performance ofاhollow corrugated tube columns compared to flat tubes, with compressive strength and ductility increasing by up to 2 times.
2. CFST columns with corrugated plates perform better, with less strength reduction and higher ductility compared to flat plates due to the less of concrete area to 50% approximately. The research also examines CFDST columns with an inner void, highlighting their distinct features.
3. The study found that incorporating internal corrugated plates in CFDST columns significantly enhanced their load-carrying capacity and ductility compared to flat plates. Among various internal plate widths, columns with a 116 mm internal width demonstrated the best performance, with a 25.3% higher strength than those with a 160 mm width and a 7.4% improvement over those with a 60 mm width.
4. Inner corrugation enhances both compressive strength and ductility, while outer corrugation reduces compressive strength compared to flat plates, similar to the behavior observed in CFST columns.
5. Outer corrugated columns are preferred in high hollow ratios due to their lower weight and good compressive strength.
6. In high χ ratios, CFDST column specimens with corrugated plates exhibited comparable ultimate compression capacities, nearly matching those of CFDST columns with flat plates. This was achieved while maintaining high ductility and reducing overall column weight.
7. The GPR model demonstrated exceptional predictive accuracy for N_exp_​, achieving an R-value of approximately 0.99 for both training and testing datasets. In comparison, the ANN model showed less effectiveness, with RMSE values of 197.21 kN and MAE values of 328.61 kN during testing, whereas the GPR model achieved lower RMSE (267.80 kN) and MAE (157.11 kN), highlighting its superior performance.
8. Regression analysis demonstrated that the slopes of the regression lines for training data were 0.9841 for GPR and 0.9827 for ANN, both close to the ideal value of 1, indicating strong alignment between predicted and experimental values. The majority of absolute errors for predictions fell within a range of ± 0.5 × 10^5 kN, with GPR producing significantly lower errors, confirming its robustness and reliability for strength prediction in practical engineering scenarios.
9. The proposed machine learning models emerged as highly accurate and reliable for predicting the strength of square CFDST column datasets, respectively. Additionally, SHAP analysis highlighted that enhancing the width and thickness of the external tube improves the performance of square CFDST columns. Conversely, increasing the section length and reducing concrete strength negatively affects the compressive strength index.

## Electronic supplementary material

Below is the link to the electronic supplementary material.

Supplementary Material 1

Supplementary Material 2

## Data Availability

All data generated or analyzed during this study, including experimental data and data collected from previous studies (with all references to the collected data cited in the manuscript), are included in this submitted article and its supplementary information files.
